# *A disintegrin and metalloproteinase with thrombospondin motifs 4*-targeted molecular magnetic resonance imaging for early detection of extracellular matrix remodeling in a porcine model of abdominal aortic aneurysm

**DOI:** 10.1016/j.jocmr.2026.102718

**Published:** 2026-03-19

**Authors:** Marie-Luise H.H. Ranner-Hafferl, Dilyana B. Mangarova, Jennifer L. Heyl, Jennifer Mein, Jana Möckel, Dirk Schnapauff, Timo A. Auer, Federico Collettini, Lisa C. Adams, David Hingst, Jan O. Kaufmann, Marcus R. Makowski, Uwe Karst, Bernd Hamm, Julia Brangsch, Avan Kader

**Affiliations:** a Charité – Universitätsmedizin Berlin, Corporate Member of Freie Universität Berlin, Humboldt-Universität zu Berlin, Department of Radiology, Berlin, Germany; b Department of Veterinary Medicine, Institute of Animal Welfare, Animal Behavior, and Laboratory Animal Science, Freie Universität Berlin, Berlin, Germany; c Berlin Institute of Health (BIH), Berlin, Germany; d Technical University of Munich, Department of Diagnostic and Interventional Radiology, Munich, Germany; e Institute of Inorganic and Analytical Chemistry, University of Münster, Münster, Germany

**Keywords:** ADAMTS4 Protein, Aortic Aneurysm, Abdominal, Extracellular Matrix, Magnetic Resonance Imaging, Molecular Imaging, Swine

## Abstract

**Background:**

Abdominal aortic aneurysm (AAA) progression is driven by extracellular matrix (ECM) proteolysis and vascular smooth muscle cell loss, processes insufficiently captured by diameter-based surveillance. *A disintegrin and metalloproteinase with thrombospondin motifs 4* (ADAMTS4) is upregulated during early ECM remodeling, making it a compelling target for molecular imaging.

**Methods:**

Eighteen female German Landrace swine were enrolled. Eight animals underwent AAA induction and four served as controls; six animals were excluded due to procedural complications. Molecular 3 T MRI was performed at two and four weeks after induction following intravenous administration of an ADAMTS4-specific gadolinium probe (∼0.03 mmol/kg). Contrast-to-noise ratio (CNR) was calculated pre- and post-contrast. Aortic diameter was monitored by serial ultrasound. *Ex vivo* analysis included immunofluorescence, Western blotting, and laser ablation-inductively coupled plasma-mass spectrometry (LA-ICP-MS). Spearman correlation was used to evaluate relationships between MRI signal and molecular markers of ECM remodeling.

**Results:**

ΔCNR increased at two weeks (3.18 ± 0.56) and four weeks (4.35 ± 0.84) relative to controls (−0.14 ± 0.57; *p* = 0.017 and *p* = 0.046). ADAMTS4 immunofluorescence increased to 43.91 ± 9.48% at two weeks and 46.97 ± 4.50% at four weeks (control: 0.39 ± 0.03%). ΔCNR strongly correlated with ADAMTS4 (*r*_*s*_ = 0.92, *p* < 0.001) and Galectin-3 (*r*_*s*_ = 0.87, *p* < 0.001), and inversely with alpha-smooth muscle actin (*r*_*s*_ = −0.90, *p* < 0.001), indicating that MRI signal reflects active proteolytic remodeling and smooth muscle cell depletion. LA-ICP-MS confirmed focal probe accumulation in regions of high ADAMTS4 expression.

**Conclusion:**

ADAMTS4-targeted molecular MRI enables early, non-invasive detection of ongoing ECM remodeling in AAA and provides activity-based disease characterization beyond diameter-based assessment.

## Graphical abstract

**Figure:**
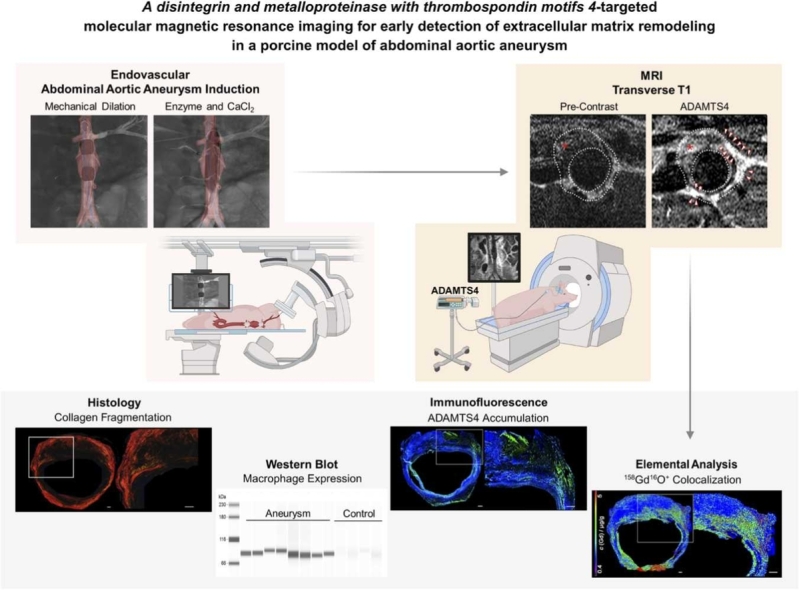
Created in BioRender.com (2026).

## 1 Introduction

Abdominal aortic aneurysm (AAA) remains a major clinical challenge due to its asymptomatic progression and high mortality upon rupture [1]. Current monitoring strategies rely on anatomical imaging to assess aortic diameter over time [2], [3]. This size-based approach detects more advanced structural changes only and fails to identify patients at the highest risk for rapid progression or rupture [4], [5].

The pathogenesis of AAA involves complex interactions between inflammatory cell infiltration, vascular smooth muscle cells (VSMC) apoptosis, and progressive degradation of essential extracellular matrix (ECM) components [6]. These molecular changes often precede observable anatomical alterations and may serve as more sensitive indicators of disease activity. Among the proteolytic enzymes driving ECM breakdown, matrix metalloproteinases (MMPs) and aggrecanases play central roles, with *a disintegrin and metalloproteinase with thrombospondin motifs 4* (ADAMTS4) emerging as a particularly promising biomarker [7], [8].

ADAMTS4 cleaves versican and other proteoglycans, essential for aortic wall integrity, and increased expression has been linked to aneurysm progression and atherosclerotic plaque instability in both human tissue and experimental models [9]. Unlike constitutively expressed structural proteins, ADAMTS4 is dynamically upregulated specifically in regions of active ECM remodeling, making it a potential target for molecular imaging approaches.

Recent advances in molecular MRI have demonstrated the feasibility of visualizing proteins and enzyme activity *in vivo* using targeted contrast probes [10]. Kaufmann *et al.* (2022) successfully developed an ADAMTS4-specific gadolinium-bound probe that achieved high sensitivity and specificity in murine AAA models, with signal enhancement preceding measurable diameter changes [11]. These advances suggest the potential to move AAA diagnostics beyond size alone, toward biologically meaningful imaging biomarkers that reflect rupture risk [12].

Large animal models, particularly pigs, closely resemble human aortic anatomy, hemodynamics, and ECM composition. Porcine abdominal aortic wall thickness at physiological pressure typically ranges from 0.5 to 1 mm, depending on segment and loading conditions, which corresponds well with scaled human infrarenal aortic dimensions in middle-aged individuals [13]. Moreover, the elastic compliance and circumferential stiffness of young pig aortic tissue are comparable to those of healthy human aortas under 100 mmHg, making porcine models highly relevant for AAA imaging and device-testing studies [14], [15].

To date, no studies have evaluated ADAMTS4-targeted molecular MRI in a large animal model of AAA. This study addresses that gap by evaluating an ADAMTS4-specific gadolinium-bound MRI contrast probe in an endovascular swine model, which may offer a more sensitive and specific approach for monitoring AAA progression than current imaging standards.

We hypothesized that ADAMTS4-targeted molecular MRI would directly detect proteolytic ECM remodeling *in vivo*, enabling early identification of biologically active AAA beyond diameter-based metrics.

## 2 Methods

### 2.1 Animals

All *in vivo* procedures were conducted in compliance with the directive 2010/63/EU of the European Parliament on the protection of animals used for scientific purposes and were approved by the State Office for Health and Social Affairs of Berlin (G 0077/21). The Animal Research: Reporting of In Vivo Experiments (ARRIVE) guidelines [16] were followed and 3^R^ (Replacement, Refinement, Reduction) principles were applied wherever possible to promote ethical animal experimentation.

**Fig. 1:**
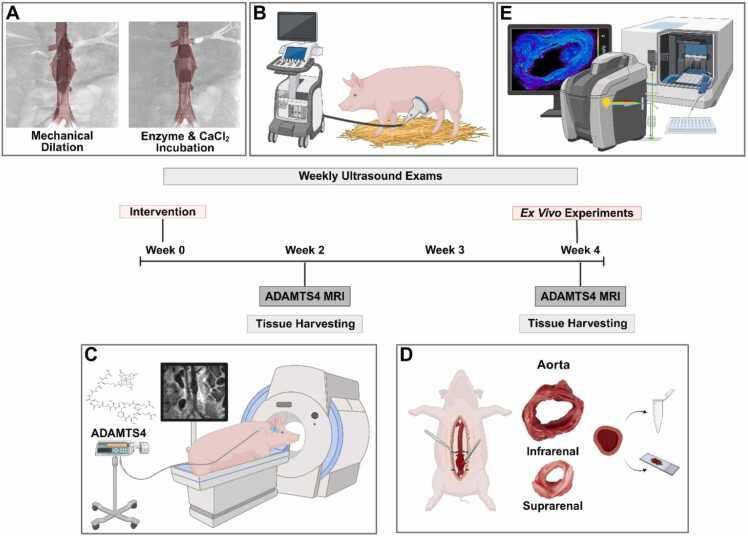
Study design. **A**, Abdominal aortic aneurysm induction via mechanical dilation, endoluminal elastase, collagenase, and calcium chloride incubation. **B**, Weekly ultrasound examinations monitored aortic diameter progression. **C**, MRI was performed at two and four weeks (2 W, 4 W) post-intervention using a gadolinium-bound ADAMTS4-specific molecular probe; control animals (*n* = 4) were imaged post-acclimatization. **D**, Tissue was harvested at 2 W and 4 W for gross aortic diameter measurements. **E**, *Ex vivo* analyses included histology, immunofluorescence, Western blot, and laser ablation-inductively coupled plasma-mass spectrometry. *MRI* magnetic resonance imaging

The study included 18 female German Landrace swine (*N* = 18; 30–40 kg). Eight animals completed the protocol and were randomized for MRI follow-up at two weeks (2 W; *n* = 4) or four weeks (4 W; *n* = 4) post-intervention. Four additional animals served as healthy controls (*n* = 4) and were imaged post-acclimatization (Fig. 1, study design).

Animals were acclimatized for three weeks prior to intervention at the Research Facilities for Experimental Medicine, Charité – Universitätsmedizin Berlin. All interventional procedures were performed by interventional radiologists with more than five years of experience.

### 2.2 Endovascular interventional protocol

The interventional procedures, including anesthesia, vascular access, AAA induction via balloon dilation, enzymatic degradation, calcium chloride incubation, postoperative care, and ultrasound monitoring, were performed as previously described [17]. In brief, animals underwent general anesthesia, infrarenal aortic dilation using a 14 × 40 mm balloon at 6–8 bar for ten minutes, followed by Fogarty catheter occlusion, enzymatic incubation with collagenase and elastase (6000 I.U. and 500 I.U., respectively; 20 min), and calcium chloride (25%, 0.5 mL; 15 min) before angiographic confirmation of technical success. Puncture sites were closed using Angio-Seal devices. Postoperative care included analgesia, antibiotics, and frequent clinical monitoring by veterinarians.

### 2.3 Ultrasound examinations

Ultrasound procedures are described in the Supplementary Material.

### 2.4 ADAMTS4-specific gadolinium-bound probe specifications

Detailed molecular ADAMTS4-probe manufacturing specifications are described in the Supplementary Material*.*

### 2.5 MR imaging procedure

Detailed MR imaging protocols, including individual sequence acquisition parameters are described in the Supplementary Material.

### 2.6 MR imaging analysis

MR imaging analysis is described in the Supplementary Material.

### 2.7 Tissue harvest and *ex vivo* analyses

Tissue harvesting and *ex vivo* analyses are described in detail in the Supplementary Material.

### 2.8 Histopathology

Histology staining procedures are described in the Supplementary Material*.*

### 2.9 Immunofluorescence and Western blotting

Experimental protocols for immunofluorescence (IF) staining and Western blotting (WB) are described in the Supplementary Material*.*

### 2.10 Laser ablation-inductively coupled plasma-mass spectrometry

Laser ablation-inductively coupled plasma-mass spectrometry (LA-ICP-MS) is described in the Supplementary Material.

### 2.11 Statistical analyses

Statistical analyses were conducted using IBM SPSS Statistics (Version 29.0.0; IBM Corp., Armonk, New York). Graphs and visualizations were created using GraphPad Prism (Version 10.4.1; Dotmatics, Boston, Massachusetts). Figures and schematics were produced using BioRender (2025; Science Suite Inc., Toronto, Ontario, Canada).

Data are presented as mean ± standard deviation. Normality of data distributions was assessed using the Shapiro-Wilk test, and homogeneity of variances using Levene’s test. For normally distributed data, one-way analysis of variance (ANOVA) was performed with Tukey’s or Dunnett’s T3 post hoc testing as appropriate. For non-normally distributed data, Brown-Forsythe or Welch ANOVA was applied, followed by Games-Howell post hoc testing where required.

Pairwise associations of imaging metrics and molecular markers were assessed using two-tailed Spearman's rank correlation analysis. Correlation coefficients (*r*_*s*_) are reported together with corresponding *p*-values and 95% confidence intervals. Statistical significance was defined as α < 0.05.

## 3 Results

**Fig. 2:**
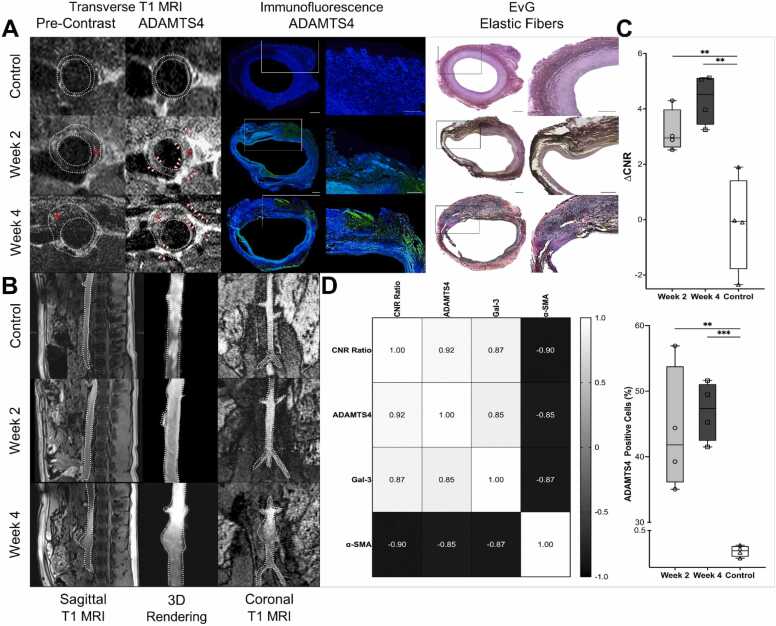
*In vivo* molecular MRI visualization of ADAMTS4 in porcine aneurysmal abdominal aortas. **A**, Transverse T1-weighted MR images of the infrarenal aorta before contrast and following administration of the ADAMTS4-targeted gadolinium probe, with corresponding ADAMTS4 immunofluorescence (green; DAPI, blue) and EvG elastic-fiber staining shown for control, two week (2 W), and four week (4 W) aneurysms. Arrowheads (∆) indicate probe-enhanced regions within the aortic wall; asterisks (*) denote intraluminal thrombus. White dashed lines outline vessel boundaries. **B**, Sagittal T1 MRI, 3D vessel rendering, and coronal T1 MRI reconstructions illustrating progressive aneurysmal remodeling across timepoints. **C**, Top: Quantification of change in contrast-to-noise ratio (∆CNR) demonstrating significantly increased post-contrast signal enhancement in aneurysmal aortas at 2 W and 4 W compared to controls. Bottom: Quantification of ADAMTS4-positive cells by immunofluorescence confirming increased ADAMTS4 expression in aneurysmal tissue at 2 W and 4 W relative to controls. Individual values: *n* = 4 for 2 W (dots); *n* = 4 for 4 W (squares); *n* = 4 for controls (triangles). **D**, Spearman correlation matrix showing relationships between ΔCNR and extracellular matrix remodeling markers. ΔCNR correlates positively with ADAMTS4 and Galectin-3 and negatively with alpha-smooth muscle actin, indicating increased proteolytic remodeling and reduced contractile smooth muscle phenotype during aneurysm progression*.* Scale bars: 500 µm. Statistical significance: **p < 0.01, ***p < 0.001. *EvG* Elastica van Gieson, *ADAMTS4* a disintegrin and metalloproteinase with thrombospondin motifs 4.

The experimental protocol produced porcine infrarenal aneurysms that progressed over time, allowing detailed assessment of both anatomical and molecular disease features. Five animals required early euthanasia due to aortic rupture (*n* = 2) or hind-limb paresis (*n* = 3). An additional animal did not develop an aneurysm because of a technical failure during intervention and was euthanized at the study endpoint (*n* = 1). MRI revealed characteristic changes in gross vessel morphology, while molecular MRI enabled *in vivo* visualization of ADAMTS4 expression (Fig. 2B). Weekly ultrasound examinations following the AAA induction procedure (baseline: 0.74 ± 0.08 cm) revealed progressive diameter growth. By week two, the mean aortic diameter was 1.59 ± 0.06 (+107.14 ± 4.04% vs. day 0; *p* < 0.001), by week four, measurements showed 1.94 ± 0.19 (+161.03 ± 15.77% vs. day 0, *p* = 0.002), as previously described [17].

### 3.1 MRI signal enhancement

T1-weighted molecular MRI using the ADAMTS4-specific gadolinium-bound contrast probe revealed a distinct increase in signal intensity within the aneurysmal wall compared to native T1 images (Fig. 2A). At 2 W, the CNR rose from 8.90 ± 1.73 to 12.08 ± 1.73 (ΔCNR = 3.18 ± 0.56; *p* = 0.017 vs. control), and at 4 W from 6.29 ± 1.34 to 10.64 ± 0.86 (ΔCNR = 4.35 ± 0.84; *p* = 0.046 vs. control). In control animals, no significant signal enhancement was observed (pre-contrast CNR: 8.03 ± 1.10; post-contrast CNR: 7.89 ± 1.23; ΔCNR: −0.14 ± 0.57) (Fig. 2C).

### 3.2 MRI signal enhancement and extracellular matrix degradation correlation

Spearman correlation analysis revealed strong associations between MRI contrast changes and molecular remodeling markers (Fig. 2D).

Pairwise analysis revealed strong positive associations between ΔCNR and both ADAMTS4 (*r*_*s*_ = 0.92, *p* = 0.00008, 95% CI: 0.71–0.98) and Galectin-3 (*r*_*s*_ = 0.87, *p* = 0.0004, 95% CI: 0.59–0.97). In contrast, ΔCNR demonstrated a strong inverse correlation with alpha-smooth muscle actin (α-SMA) expression (*r*_*s*_ = –0.90, *p* = 0.0002, 95% CI: –0.97 to –0.67). ADAMTS4 and Gal-3 were also positively correlated (*r*_*s*_ = 0.85, *p* = 0.0009, 95% CI: 0.52–0.96), whereas both ADAMTS4 and Gal-3 showed strong negative correlations with α-SMA (*r*_*s*_ = –0.85 and –0.87, respectively; both *p* < 0.001).

In addition to rank-based correlation analysis, linear regression confirmed a significant positive association between ΔCNR and ADAMTS4-positive area (*R*² = 0.70, *F*(1,10) = 23.84, *p* = 0.0006; *Table S-II*), indicating that approximately 70% of the variance in MRI signal enhancement can be explained by ADAMTS4 expression. Together, these analyses indicate that increased MR signal enhancement is associated with increased ECM-remodeling activity and loss of contractile smooth muscle phenotype.

### 3.3 *Ex vivo* ADAMTS4 quantification

**Fig. 3:**
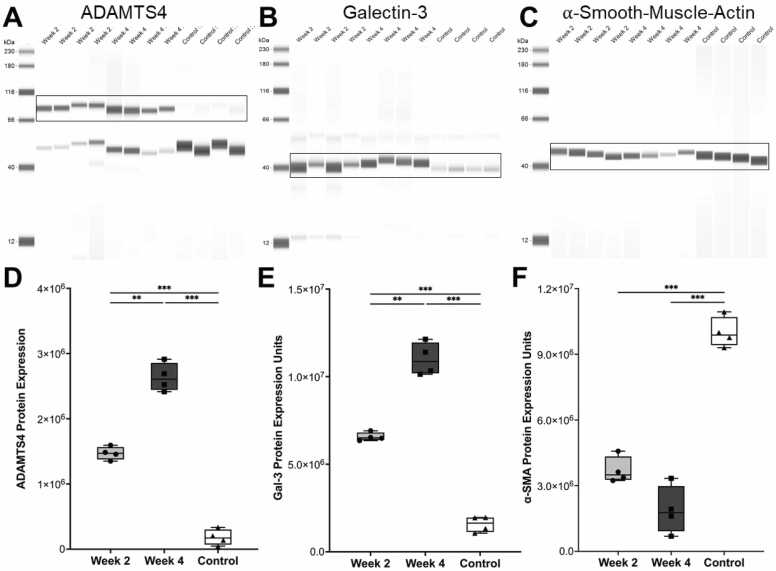
*Ex vivo* quantification of ADAMTS4, Galectin-3, and alpha-smooth muscle actin expression in aneurysmal and healthy porcine aortas. **A–C**, Representative Western blot bands for ADAMTS4 (∼90 kDa full-length and ∼45 kDa processed forms), Galectin-3 (Gal-3; ∼31 kDa), and alpha-smooth muscle actin (α-SMA; ∼42 kDa) from two weeks (2 W) post-AAA induction, four weeks (4 W) post-AAA induction, and control animals. **D–F**, Densitometric quantification reveals progressive ADAMTS4 upregulation (control: 6.22 ± 4.13%, 2 W: 50.52 ± 3.43%, 4 W: 90.51 ± 7.44%; *p* < 0.001), increased Gal-3 expression (control: 12.98 ± 3.70%, 2 W: 54.12 ± 1.97%, 4 W: 90.65 ± 7.72%; *p* < 0.001), and reduced α-SMA expression (control: 91.43 ± 6.29%, 2 W: 33.82 ± 5.54%, 4 W: 17.28 ± 10.02%; *p* < 0.001) in AAA tissue. Individual values: *n* = 4 for 2 W (dots); *n* = 4 for 4 W (squares); *n* = 4 for controls (triangles). Statistical significance: **p < 0.01, ***p < 0.001 *ADAMTS4* a disintegrin and metalloproteinase with thrombospondin motifs 4, *AAA* abdominal aortic aneurysm.

WB demonstrated a progressive increase in ADAMTS4 expression in aneurysmal tissue from 50.52 ± 3.43% for animals euthanized 2 W after AAA induction, and 90.51 ± 7.44% at 4 W (control: 6.22 ± 4.13%, *p* < 0.001) (Figs. 3A, 3D).

IF analysis confirmed these results, showing ADAMTS4 localized to the medial and adventitial layers, with 43.91 ± 9.48% (*p* = 0.006) of the total aortic area showing ADAMTS4-positive cells at 2 W; this increased to 46.97 ± 4.50% (*p* < 0.001) at 4 W (control: 0.39 ± 0.03%) (Figs. 2A, 2C).

**Fig. 4:**
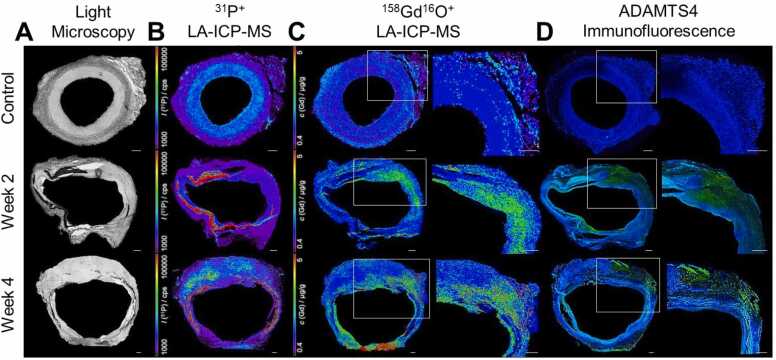
Spatial correlation of gadolinium deposition with ADAMTS4 expression by laser ablation-inductively coupled plasma-mass spectrometry. **A**, Representative bright field light microscopy cross-sections of infrarenal aorta from control animals, two weeks (2 W) after AAA induction, and four weeks (4 W) post-AAA induction. **B**, ^31^P^+^ LA-ICP-MS elemental maps showing overall tissue distribution, **C**, ^158^Gd^16^O^+^ LA-ICP-MS maps indicating gadolinium localization following administration of the ADAMTS4-targeted probe, **D**, ADAMTS4 immunofluorescence (green) with nuclear DAPI counterstain (blue). AAA tissue at 2 W and 4 W shows focal gadolinium accumulation co-localizing with ADAMTS4-positive regions, whereas control tissue exhibits negligible gadolinium signal. Scale bars: 500 µm. *AAA* abdominal aortic aneurysm, *LA-ICP-MS* laser ablation-inductively coupled plasma-mass spectrometry, *ADAMTS4* a disintegrin and metalloproteinase with thrombospondin motifs 4.

These regions of antibody binding correlated spatially with focal gadolinium deposition patterns observed by LA-ICP-MS, which visually confirmed gadolinium-bound probe accumulation in regions with high ADAMTS4-positive cell density by IF (Fig. 4).

### 3.4 Vascular inflammation and endothelial remodeling

Gal-3 WB revealed increased macrophage infiltration in both intervention groups (2 W: 54.12 ± 1.97%, *n* = 4; 4 W: 90.65 ± 7.72%, *n* = 4) compared to controls (12.98 ± 3.70%, *n* = 4, *p* < 0.001). α-SMA expression, indicative of VSMC content, was determined to be depleted in treated swine. WB quantification revealed α-SMA protein levels of 33.82 ± 5.54% and 17.28 ± 10.02% in the 2 W and 4 W groups, respectively, compared to 91.43 ± 6.29% in controls (*p* < 0.001) (Fig. 3B, C, E, F).

**Fig. 5:**
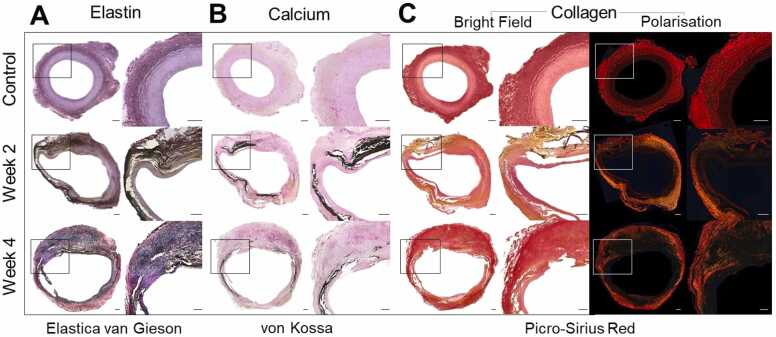
Histopathological features of abdominal aortic aneurysms and healthy aortic tissue. Representative cross-sections of infrarenal aorta from control, two weeks (2 W) after AAA induction, and four weeks (4 W) post-intervention. **A**, Elastica-van-Gieson staining for elastin: control shows intact elastic lamellae, whereas 2 W and 4 W AAA sections reveal progressive fragmentation and loss of elastic fibers. **B**, Von Kossa staining for calcium: no deposition in control tissue, with focal to extensive medial calcification in AAA tissue. **C**, Picro-Sirius Red staining for collagen visualized under bright field (left) and polarized light (right): control shows organized, densely packed collagen fibers, while AAA tissue demonstrates disrupted, disorganized collagen architecture at 2 W and 4 W. Scale bars: 500 µm. *AAA* abdominal aortic aneurysm

Histopathological analysis revealed fragmentation of elastic and collagen fibers, along with calcium deposition, mirroring changes typically observed in human AAA (Fig. 5).

## 4 Discussion

This study demonstrates that ADAMTS4-targeted molecular MRI can detect active ECM remodeling in a porcine AAA model. The observed increase in CNR following probe administration, relative to pre-contrast T1-weighted MRI at two, and four weeks post-aneurysm induction, was corroborated by *ex vivo* ADAMTS4 quantification via protein assays, immunostaining, and spatial gadolinium mapping by LA-ICP-MS, while healthy control animals showed no significant post-contrast signal increase. Histopathology revealed characteristic fragmentation of elastin and collagen fibers and localized calcium deposition in the porcine aneurysmal wall, mirroring hallmarks of human AAA pathogenesis (Fig. 5).

These findings validate both the probe’s specificity and the sensitivity of the imaging approach. Moreover, they extend the applicability of ADAMTS4-targeted imaging from murine systems to a clinically relevant large animal model, highlighting its translational potential.

### 4.1 ADAMTS4 as a valuable molecular target for AAA imaging

Challenging conventional imaging biomarkers, ADAMTS4 exhibits several characteristics that make it particularly well suited for AAA molecular imaging. First, ADAMTS4 shows temporal specificity, being upregulated during active disease rather than constitutively. Second, by indexing ongoing enzymatic degradation, it serves as a mechanistic marker of progression, whereas structural targets (e.g., elastin, collagen) primarily reflect end-stage damage [18]. Third, unlike broadly expressed inflammatory markers, ADAMTS4 provides greater specificity for the ECM-degrading processes characteristic of aneurysmal disease and is selectively upregulated in regions of active ECM remodeling and inflammation [19], [20], [21]. Our data support this concept, as ADAMTS4 expression increased progressively, directly correlating with the progressive nature of aneurysmal changes. The temporal correlation between ADAMTS4 upregulation and disease severity, confirmed by both molecular MRI and biochemical quantification, validates its role as a dynamic disease biomarker [22]. This approach enables improved biological characterization of AAAs beyond conventional size-based measurements or static molecular targets, including the assessment of disease activity and monitoring of therapeutic response [23].

The mechanistic role of ADAMTS4 in AAA pathogenesis is well-established. Novak *et al*. (2022) demonstrated that ADAMTS4 degrades versican and other proteoglycans, essential for vessel wall integrity, leading to ECM disorganization and enhanced inflammatory cell infiltration [24]. This proteolytic activity creates a self-perpetuating cycle where ECM degradation facilitates further inflammation, which in turn upregulates additional ADAMTS4 expression. Yuan *et al.* (2024) described identifying neutrophil subtypes with high ADAMTS/ADAM protease expression, further emphasizing their role in inflammatory signaling within the aneurysmal microenvironment [25]. Dale *et al.* (2015) highlighted the importance of therapeutic strategies targeting macrophage phenotypes in order to decelerate aneurysm growth, which involves modulation of ADAMTS4-expression patterns [26]. Collectively, these mechanistic insights support ADAMTS4 as an empirically validated molecular imaging target that captures the active, destructive remodeling processes characteristic of AAA progression, distinguishing it from passive structural changes detected by conventional imaging [27].

### 4.2 Complementary large animal MRI studies

While this study focused on ADAMTS4-specific gadolinium‑bound probes, it is important to contextualize the findings within the broader landscape of molecular imaging approaches for AAA. Ultrasmall superparamagnetic iron-oxide nanoparticles (USPIO) represent an alternative strategy, targeting macrophage-driven inflammation through T2*/T2-weighted MRI [28]. In clinical-scale models of aortic aneurysm and atherosclerosis, USPIO uptake co-localized with macrophage infiltration, and the scale of signal change correlated with the extent of inflammatory activity and progression risk [29], [30]. For instance, the MA^3^RS study in humans, and experimental aneurysms in large animals, showed high USPIO retention in rapidly expanding AAAs with a strong correlation of macrophage marker quantity [31].

However, USPIO-based approaches detect general inflammatory activity rather than specific enzymatic processes. As demonstrated by the spatial correlation between gadolinium deposition and ADAMTS4 expression patterns observed through LA-ICP-MS analysis, the ADAMTS4-specific imaging approach offers more specific molecular information. By enabling visualization of distinctly localized ADAMTS4 activity rather than a mere confirmation of upregulation in the whole infrarenal aortic wall, better discrimination between different types of vascular pathologies and more precise monitoring of ECM-targeted therapies may be facilitated. Positron emission tomography (PET)-based tracers such as ^64^Cu-Macrin for macrophage tracking, have demonstrated feasibility in large animal models [32]. However, these approaches share the limitation of targeting general inflammatory processes rather than specific degradative enzymes. The advantage of ADAMTS4-targeted imaging lies in its ability to detect the specific enzymatic activity responsible for ECM degradation, potentially providing more precise and clinically significant information.

### 4.3 Extracellular matrix-stabilizing therapies as an ADAMTS4 monitoring use case

The specific enzymatic activity of ADAMTS4 in cleaving versican, aggrecan and other proteoglycans, together with preclinical evidence, demonstrates that ADAMTS4 modulation has direct therapeutic effects [33]. Genetic deletion or miR-126a-5p-mediated downregulation reduces aneurysm progression in rodent models, establishing a clear link between ADAMTS4 activity and structural preservation [34], [35]. This supports the use of ADAMTS4-targeted imaging as a pharmacodynamic biomarker for evaluating therapeutic efficacy, potentially enabling earlier assessment of treatment responses than conventional anatomical endpoints.

Furthermore, the novel, purely endovascular porcine model of AAA used as a vehicle in this study, successfully reproduced key pathophysiological features of human AAA, as evidenced by characteristically progressive histopathological changes including elastic-fiber fragmentation, collagen disorganization, and calcium deposition [35], [36]. The use of clinical-grade 3 T MRI scanners and standard contrast sequencing protocols further enhances translational relevance of this molecular imaging project [37].

### 4.4 Monitoring of protease-targeted and anti-inflammatory therapies

Therapeutic approaches targeting the proteolytic cascade show promise for AAA treatment. For instance, broad MMP inhibition with doxycycline attenuates aneurysm expansion and preserves elastin in rodents, and porcine aortic organoids show elastic-fiber perpetuation with reduced MMP-9 activity [38], [39].

By linking ECM-focused therapies to a measurable molecular biomarker, ADAMTS4-targeted MRI in large-animal models could enable early therapy efficacy assessment and dose optimization, potentially strengthening translation toward clinical use.

## 5 Limitations

This study’s cross-sectional design with terminal endpoints at discrete timepoints prevented longitudinal assessment of individual animals, limiting insights into temporal progression patterns and inter-individual variability in disease development. Future longitudinal studies tracking individual animals over time will be essential for validating the predictive value of ADAMTS4-imaging for disease progression and rupture risk assessment.

The small sample size and relatively high drop-out rate limit statistical robustness and generalizability of findings. Dropouts in the initial project-phase reflected procedural errors in the AAA induction. Once interventional, anesthetic, and ventilatory parameters were adjusted and standardized, drop-out rates fell and remained low, indicating the procedure itself is not inherently high risk. The ADAMTS4 administration produced no adverse reactions.

Additionally, the study was restricted to female animals because bladder catheterization in male pigs was not feasible. Follow-up projects utilizing male swine are planned as warranted by the sex dimorphism of AAA disease.

## 6 Conclusion

ADAMTS4-targeted MRI in a porcine AAA model enables early, non-invasive detection of ECM remodeling before severe expansion. This molecular biomarker may support risk stratification and therapeutic monitoring, warranting longitudinal and translational studies.

## Funding

This research project was funded by the Deutsche Forschungsgemeinschaft (DFG, German Research Foundation) − Project-ID 372486779 − SFB 1340, B01.

## Author contributions

**Marie-Luise H.H. Ranner-Hafferl:** Writing – review & editing, Writing – original draft, Visualization, Investigation, Formal analysis, Data curation. **Dilyana B. Mangarova:** Writing – review & editing, Investigation. **Jennifer L. Heyl:** Writing – review & editing, Investigation, Formal analysis. **Jennifer Mein:** Writing – review & editing, Investigation. **Jana Möckel:** Writing – review & editing, Investigation. **Dirk Schnapauff:** Writing – review & editing, Methodology, Investigation. **Timo A. Auer:** Writing – review & editing, Methodology, Investigation. **Federico Collettini:** Writing – review & editing, Supervision, Methodology, Investigation, Funding acquisition. **Lisa C. Adams:** Writing – review & editing, Supervision, Resources, Funding acquisition. **David Hingst:** Writing – review & editing, Validation, Resources. **Jan O. Kaufmann:** Writing – review & editing, Resources, Methodology. **Marcus R. Makowski:** Writing – review & editing, Supervision, Resources, Funding acquisition, Conceptualization. **Uwe Karst:** Writing – review & editing, Validation, Resources. **Bernd Hamm:** Writing – review & editing, Supervision, Resources, Project administration, Funding acquisition, Conceptualization. **Julia Brangsch:** Writing – review & editing, Supervision, Project administration, Methodology, Investigation, Funding acquisition, Formal analysis, Conceptualization. **Avan Kader:** Writing – review & editing, Supervision, Project administration, Methodology, Investigation, Funding acquisition, Conceptualization.

## Ethics approval and consent

All animal experiments were performed in accordance with the German Animal Welfare Act and the European Directive 2010/63/EU for the protection of animals used for scientific purposes. The study was approved by the State Office of Health and Social Affairs Berlin (LAGeSo Berlin) under the approval number G 0077/21. Approval was granted on 05 November 2021

## Declaration of competing interests

The authors declare that they have no known competing financial interests or personal relationships that could have appeared to influence the work reported in this paper.

## Data Availability

The datasets generated during the current study are available from the corresponding author upon reasonable request.
