# Supplementary material for: A disintegrin and metalloproteinase with thrombospondin motifs 4-targeted molecular magnetic resonance imaging for early detection of extracellular matrix remodeling in a porcine model of abdominal aortic aneurysm
Source: J Cardiovasc Magn Reson. 2026 Mar 19;28(1):102718. doi: 10.1016/j.jocmr.2026.102718 (PMC13237536; doi:10.1016/j.jocmr.2026.102718)
Supplement: Supplementary file 1 — Supplementary material [file mmc1.docx]

**Supplementary Methods**

**Ultrasound Examinations**

Following Ranner-Hafferl *et al.* (2026), scans were performed pre-intervention and weekly to monitor AAA growth and rupture risk. Imaging used an ArtUs EXT-2H with a C5-2H60-A5 transducer (Telemed Ltd., Vilnius, Lithuania) and Echo Wave II (Version 4.2.0, 64-bit; Telemed Ltd.) [1]. Baseline and terminal scans were under sedation; interim follow-ups were in awake, habituated animals. Standard settings were ~120 mm depth, 72 dB dynamic range, 48–50 fps, and 5 MHz. The probe was placed on the right flank ~5 cm cranial to the anterior superior iliac spine; the right kidney standardized planes before visualizing the abdominal aorta and inferior vena cava. If colonic gas impeded imaging, additional pressure was applied or the left flank was used. At each time point, three images per animal were measured in Horos software (Version 4.0.1; The Horos Project, Annapolis, MD, USA) and averaged to obtain a representative aortic diameter.

**ADAMTS4-Specific Gadolinium-Bound Probe Specifications**

The ADAMTS4-specific molecular MRI probe was synthesized by Gyros Protein Technologies AB (Teltow, BB, Germany) using a PurePep^®^ Sonata+ peptide synthesizer (Gyros Protein Technologies, Tucson, AZ, USA). Synthesis followed standard fluorenylmethyloxycarbonyl (Fmoc) solid-phase peptide synthesis (SPPS) protocols, with DOTA(tBu)_3_-OH (#F792189; Fluorochem EU Ltd., Cork, Ireland) incorporated on the lysine side chain during SPPS. PurePep EasyClean^®^-Linker RC+ (PEC; 8×25 µmol #P0020873, #P0020872 8×100 µmol) was used, with coupling restricted to full-length sequences as the final residue. After Trifluoroacetic acid (TFA) cleavage (#SOL-011; Iris Biotech GmbH, Marktredwitz, BY, Germany) and diethyl ether precipitation (#5920.2; Carl Roth GmbH, Karlsruhe, BW, Germany), the peptide was redissolved in water. The gadoteric acid (DOTA) chelate was introduced using DOTA(tBu)_3_-OH (#F792189; Fluorochem EU Ltd., Cork, Ireland), and the gadolinium was incorporated with GdCl_3_ (#AB120502; abcr GmbH, Karlsruhe, BW, Germany). Thereafter, PEC-purification was performed (RC+ workflow as described by Zitterbart *et al*., 2021), the peptide was obtained with TFA counter-ion as a diethyl ether precipitate (#5920.2; Carl Roth GmbH), redissolved in water, and converted to the chloride salt by strong-base anion exchange [1]. Disulfide formation was induced at pH 8.0 in ammonium hydroxide; after lyophilization the finished peptide was obtained as a pale yellow solid and stored at -20 °C.

Analytical quality control was conducted via Ultra-Performance Liquid Chromatography-Electrospray Ionization-Mass Spectrometry (UPLC-ESI-MS) on a Waters ACQUITY UPLC H-Class system (Waters Corp., Milford, MA, USA) with a C18 column (#186002350; Waters Corp., Milford, MA, USA) and gradient elution from 0% to 70% acetonitrile (#8825.2; Carl Roth GmbH) in seven minutes; UV absorbance was recorded at 210 nm. The probe had a calculated molecular weight of 1806.02 g/mol (C-terminal amidated 13-mer peptide) and observed m/z values matching theoretical predictions (602.8 / 903.8).

Final UV-purity of the product was 90.6%, meeting >90% PEC-grade standards.

The longitudinal relaxivity (r1) of the ADAMTS4-targeted gadolinium probe was previously characterized ex vivo at 3 T in the presence and absence of recombinant human ADAMTS4 [2]. The unbound probe demonstrated an r1 of 3.4 ± 0.19 mM−1 s−1, whereas the ADAMTS4-bound probe exhibited an increased r1 of 6.13 ± 0.73 mM−1 s−1.

This approximately twofold increase is consistent with established principles of protein-binding contrast agents, in which reduced rotational tumbling enhances relaxivity [3].

Prior to application, the ADAMTS4-GdCl_3_ peptide was dissolved in 10 mL 0.9% NaCl solution (#2350756; B. Braun Melsungen AG) and sterile-filtered using a 0.2 µm syringe filter (#SLGSR33SS; Merck Millipore, Billerica, MA, USA). The solution was administered intravenously (i.v.) via a perfusor at a controlled infusion rate of 60 mL/h.

During the process of i.v. ADAMTS4-administration, animals were mechanically ventilated and sedated by propofol anesthesia. Animals’ vital signs were continuously monitored by pulsoximetry and auscultation. The animals showed no adverse reactions to the ADAMTS4-probe, parameters remained consistent throughout. Imaging acquisition was performed immediately after the animals were repositioned in the scanner following ADAMTS4 probe application.

**MR Imaging Procedure**

Imaging was conducted using a clinical 3 Tesla fully integrated whole-body MR scanner (Siemens Biograph mMR, Siemens Healthineers AG; Erlangen, Germany) with a 60 cm bore, 45 mT/m gradients (200 T/m/s), TrueForm magnet and Total-imaging-matrix coil architecture, equipped with a resonant radiofrequency transmit/receive abdominal coil (#10606492, mMR Body Coil; Siemens Healthineers AG Erlangen, Germany). Intubated and mechanically ventilated animals were placed in supine position under i.v. propofol general anesthesia. To locate the abdominal aorta, a defined low-resolution 3D localizer was used, before axial, sagittal, and coronal T1-weighted sequence acquirement. Localizer parameters were field-of-view (FOV) 60 mm, 10 slices, slice thickness 2.0 mm; matrix 128 × 128; repetition time (TR)/echo time (TE) 4.2/1.6 ms; flip angle 10°.

*Axial T1-Weighted 2D Turbo Spin-Echo*

Matrix 256 × 256; FOV 40 mm; in-plane resolution 0.16 × 0.16 mm; slice thickness 0.5 mm (three consecutive slices used for analysis); TR/TE 700/10 ms; echo train length 4; bandwidth ~250 Hz/px; fat suppression spectral attenuated inversion recovery; acceleration generalized auto calibrating partially parallel acquisitions (GRAPPA) factor 2. Pre-contrast and post-contrast (after ADAMTS4-specific gadolinium-bound probe) datasets were acquired with identical geometry.

*T1-Weighted 3D Spoiled Gradient-Echo*

Water-only reconstruction was used for wall contrast. Matrix 416 × 416; FOV 60 × 40 mm; voxel 0.18 mm^3^ isotropic; TR 4.2 ms; TE1/TE2 1.3/2.6 ms; flip angle 12°; bandwidth ≥ 440 Hz/px; acceleration GRAPPA factor 2. Axial source images with coronal/sagittal multiplanar reformats were stored.

To optimize blood signal nulling for late enhancement of the ADAMTS4-specific probe, a 2D Look-Locker inversion-recovery sequence was performed at the analysis level: matrix 192 × 192; FOV 40 mm; in-plane resolution 0.21 × 0.21 mm; slice 1.0 mm; inversion time (TI) sampling across 10–12 time points; readout TR/TE 8.0/3.5 ms; flip angle 12°; ~1000 ms between inversion pulses. The TI yielding blood signal nulling was used for the late-enhancement acquisition.

Finally, a 3D inversion-recovery gradient-echo late gadolinium-enhanced sequence was acquired to visualize probe uptake: FOV 50 mm; matrix 384 × 384; slice thickness 0.30 mm; 56–64 slices; in-plane resolution 0.13 × 0.13 mm; TR/TE 12/5.5 ms; TI set from Look-Locker; ~1000 ms between inversion pulses; flip angle 25°. Maximum intensity projection images were generated when useful for visualization.

All acquisition parameters and slice prescriptions were kept identical across time points and animals.

**Magnetic Resonance Imaging Analysis**

MRI analysis was performed using Horos software (Version 4.0.1, The Horos Project, Annapolis, MD, USA). CNR and ΔCNR calculations were performed exclusively on the axial 2D T1-weighted TSE sequence. Three blinded reviewers evaluated three consecutive cross-sectional images per animal. Signal intensities were measured in regions of interest (ROIs) placed over the aortic vessel wall, blood pool, and background, both before and after administration of ADAMTS4-specific gadolinium-bound probe. The contrast-to-noise ratio (CNR) was calculated by subtracting the signal intensity of the blood ROI from that of the vessel wall ROI and dividing the result by the standard deviation of pixel intensity in a background region (an ROI placed outside the animal’s anatomy on the same image). Change in CNR (ΔCNR) was calculated by subtracting the pre-contrast CNR from the post-contrast CNR (following ADAMTS4-specific gadolinium-bound probe administration) for each region of interest.

$$\mathrm{CNR}=\left( \bar{I_{\mathrm{wall}}}-\bar{I_{\mathrm{blood}}} \right)/\sigma_{\mathrm{bg}}$$

$$\Delta CNR=CNR_{post}-CNR_{pre}$$

Grubbs outlier test (*α* = 0.05) was performed on individual measurements; two values were removed and remaining values were averaged per animal.

**Tissue Harvest and *Ex Vivo* Analyses**

Animals were euthanized at 2W or 4W post-intervention under deep propofol anesthesia, followed by administration of pancuronium and potassium chloride. The aorta was dissected from the trifurcation to the suprarenal segment, flushed with phosphate-buffered saline (PBS pH 7.4, #P4417; Sigma-Aldrich^®^, Darmstadt, Germany). Infrarenal tissues sections were either embedded in optimal-cutting-temperature compound (#4583, Tissue-Tek^®^ O.C.T.^TM^, Sakura Finetek USA Inc., Torrance, CA, USA) and stored at -80 °C, fixed in formaldehyde substitute (MorFFFix^®^, #13616, MORPHISTO GmbH, Frankfurt am Main, Germany) for paraffin embedding, or frozen at -20 °C for protein analysis. Tissue harvesting, histological processing, immunofluorescence (IF), and western blotting (WB) were conducted as described by Ranner-Hafferl *et al.* (2026), with the additional use of an ADAMTS4-specific antibody (#ab185722; abcam, Cambridge, United Kingdom) for both IF and WB analyses [1].

*Histopathology*

Paraffin-embedded 10 µm sections were stained using Elastica-van-Gieson staining protocol (#12739, MORPHISTO GmbH, Offenbach, Germany) to assess elastin, Picro-Sirius red stain (#13425, MORPHISTO GmbH) to visualize collagen, and Von-Kossa (#ab150687; Abcam Ltd., Cambridge, United Kingdom) staining to evaluate calcium deposits. Microscopic imaging and morphometric analysis were conducted on stitched 10× magnification images using a Keyence BZ-X800 microscope (Keyence Corp., Osaka, Japan) and ImageJ software (Version 1.54f; National Institutes of Health, Bethesda, MD, USA). Quantified parameters included aortic diameter, luminal and external circumferences, wall thickness, and aortic area.

*Immunofluorescence and Western Blotting*

For IF, acetone-fixed (seven minutes) 10 µm cryosections were incubated overnight with primary antibodies diluted with antibody diluent (#S3022; Agilent Technologies, Santa Clara, CA, USA) against ADAMTS4 (rabbit anti-mouse monoclonal, 1:100, ab185722; Abcam Ltd.). Following primary incubation, Alexa Fluor™ 647-conjugated secondary antibodies, goat anti-mouse IgG (#A-21236, 1:1000), and donkey anti-rabbit IgG (#A-31573, 1:1000; Invitrogen, Thermo Fisher Scientific, Waltham, MA, USA) were applied and incubated once more for two hours. Sections were washed in PBS twice prior to and following primary incubation as well as before counterstaining with DAPI (#HP19.1; Carl Roth GmbH) and visualization using a Keyence BZ-X800 fluorescence microscope. Positive staining for Galectin-3 (Gal-3) and α-smooth-muscle-actin (α-SMA) was quantified by Keyence Hybrid Cell Count Software (Version 1.1.1.8; Keyence Corp.) as the proportion of stained area relative to total tissue area.

WB was performed using the Jess^TM^ Simple Western system (ProteinSimple^TM^ Biotechne Corp., San Jose, CA, USA) on protein lysates from infrarenal segments using frozen specimen lysed in 10% SDS, 1M Tris-HCl, 7M urea and glycerol (#2326.1, #9090.2, #2317.1; Carl Roth GmbH; #G5516; Sigma-Aldrich^®^), supplemented with a protease/phosphatase inhibitor cocktail (#78440; Thermo Fisher Scientific Inc.) and buffer. Following a bicinchoninic acid assay (#23227; Thermo Fisher Scientific Inc.), lysates were normalized to a final concentration of 1.25 mg/mL, denatured and loaded into 12–230 kDa capillaries (#SM-W001; ProteinSimple^TM^ Biotechne Corp., Minneapolis, MN, USA).

Chemiluminescent detection was performed with anti-mouse or anti-rabbit detection modules (#DM-002 & #DM-001; ProteinSimple^TM^ Biotechne Corp., Minneapolis, MN, USA) using Compass for Simple Western software (Version 7.0.0, ProteinSimple^TM^ Biotechne Corp., San Jose, CA, USA), and total protein normalization was achieved using the RePlex^TM^ module (#RP-001; ProteinSimple^TM^ Biotechne Corp., Minneapolis, MN, USA).

Gal-3 (rabbit polyclonal, 1:100, #14979-1-AP; Proteintech, Rosemont, IL, USA), α-SMA (mouse monoclonal, 1:100, #sc-53142; Santa Cruz Biotechnology, Dallas, TX, USA), and same as IF ADAMTS4 primary antibodies were used, observed molecular weights being around ~31 kDa, ~42 kDa and ~90 kDa respectively, as described by manufacturers.

For ADAMTS4, in addition to the expected full-length ~90 kDa band, a prominent ~45 kDa band was detected. This smaller band represents a proteolytically processed, catalytically active form of ADAMTS4 generated by endogenous or autoproteolytic cleavage of the zymogen [4]. The antibody used recognizes an epitope present in both forms, hence the ~45 kDa fragment was more abundant in control animals, likely reflecting basal processing in healthy tissue, whereas aneurysm tissue shows higher ADAMTS4 expression with a shift toward less-processed forms [5].

**Laser Ablation-Inductively Coupled Plasma-Mass Spectrometry**

Laser Ablation-Inductively Coupled Plasma-Mass Spectrometry (LA-ICP-MS) analyses were performed using an imageBIO266 laser ablation system (Elemental Scientific Lasers, Bozeman, MT, USA) coupled to an iCAP TQ ICP-MS (Thermo Fisher Scientific, Bremen, Germany) equipped with a dual concentric injector (DCI; Elemental Scientific Lasers, Bozeman, MT, USA). The instrument was operated in positive ion mode at unit mass resolution. ICP operating conditions were RF power 1500 W, auxiliary gas flow 0.80 L/min, coolant gas flow 14 L/min, and carrier/transfer gas flow 1000 mL/min. Daily tuning was performed to optimize gas flows and detector settings.

Ablation was conducted in line-by-line mode with a spot size of 40 µm and a scan speed of 320 µm/s. The laser fluence was 1 J/cm^2^ at a repetition rate of 100 Hz. The following ions were monitored (20 ms dwell time each): ^158^Gd^16^O^+^ (m/z 174), ^56^Fe^16^O^+^ (m/z 72), ^66^Zn^+^ (m/z 66), ^65^Cu^+^ (m/z 65), and ^31^P^+^ (m/z 31).

Images were processed for element localization and quantification using the in-house software Imajar (Version 3.64; Robin Schmid, Münster, Germany).

For quantification of iron and gadolinium, an external calibration with matrix-matched gelatin standards was prepared. Gelatin was spiked with iron(II) sulfate and gadolinium(III) chloride to yield iron concentrations from 0.1 to 100 µg/g and gadolinium concentrations from 0.01 to 50 µg/g. Each standard was sectioned to 10 µm thickness using a Cryostar NX70 cryostat (Thermo Fisher Scientific, Waltham, MA, USA). Limits of detection (LOD) and quantification (LOQ) were calculated using the 3σ and 10σ criteria, resulting in an LOD of 0.8 µg/g and an LOQ of 2.5 µg/g for iron, and an LOD of 0.04 µg/g and an LOQ of 0.14 µg/g for gadolinium [6].

**Standardization of analysis**

In the present AAA model [1], the aneurysmal segment was anatomically predefined and reproducibly localized between the renal arteries and the lumbar branches. This standardized induction site allowed consistent identification of the affected infrarenal aortic segment across animals.

For MRI acquisition, axial slices were prescribed perpendicular to the vessel axis and centered within this predefined segment. The same anatomical landmarks were used at follow-up examinations to ensure reproducible slice positioning.

For histopathological analysis, the corresponding infrarenal aortic segment was dissected using the same anatomical boundaries. Within this limited and anatomically well-defined region, the point of maximal dilation was easily identifiable, enabling precise correlation between imaging and tissue analysis.

**Supplementary Discussion Material**

**Cross-Species Validation and Translational Aspects**

Previous research by Kaufmann, Brangsch *et al.* (2022) in Angiotensin II-infused Apolipoprotein E‑deficient mice demonstrated that increases in ADAMTS4-MRI signal precede measurable aneurysm growth and strongly correlate with rupture risk, outperforming size-based metrics [3]. In these models, imaging results aligned closely with *ex vivo* tissue-level expression and probe uptake quantification [7].

Our porcine model demonstrates similar correlations; MRI signal enhancement at two weeks corresponded with moderate ADAMTS4 expression and concurrent anatomical changes, while four-week imaging reflected further progression of both molecular and anatomical changes. The consistency of ADAMTS4 upregulation across murine and porcine models support its translational relevance, though species-specific differences in aneurysm progression patterns must be considered for perspective clinical translation.

Despite these preclinical findings, clinical trials have not confirmed doxycycline’s impact on diameter of small infrarenal AAAs while its effect in larger aneurysms remains inadequately studied [8, 9]. Local delivery (e.g., osmotic pumps, drug-eluting balloons) may achieve high aortic wall levels with limited systemic exposure, well suited to concurrent ADAMTS4-targeted MRI. Anti-inflammatory strategies (Interleukin-1β-, or tumor necrosis factor-α-blockade) limit leukocyte infiltration, reduce MMP-, and aggrecanase-activity and should lower ADAMTS4 expression [10].

Beyond lipid lowering, statins increase tissue-inhibitor-of-metalloproteinase-1 and reduce MMPs in rabbits, while cyclodextrins enhance smooth muscle survival and autophagy, each potentially detectable as early biochemical shifts by ADAMTS4 imaging [11, 12]. Novel biologics and drug-eluting devices (e.g., intraluminal pentagalloylglucose) reinforce elastic fibers and halt expansion in rodent AAA [13].

**Supplementary Reporting**

***ARRIVE Guidelines: Essential 10***

**1. Study design**

***Experimental groups***

**AAA induction: n=8**

- Endovascular balloon dilation + intraluminal elastase/collagenase + CaCl₂.
  - n=4 imaged at **two weeks.**
  - n=4 imaged at **four weeks.**

**Controls:** n=4

- Untreated, healthy age matched animals, euthanized after MRI.

**Excluded due to initial study phase complications:** **n=6**

- **Rupture** n=2, **hindlimb paresis** n=3, **non-response** n=1.

**Experimental unit:** Individual animal (swine); held in groups of three.

**Primary objective:** Feasibility of **ADAMTS4-targeted molecular MRI** to detect early ECM remodelling in porcine AAA.

**Endpoints:** In-vivo MRI (ΔCNR) at 2W or 4W; ex-vivo tissue analyses at terminal time points.

**2. Sample size**

**Total animals used:** N=18 female German Landrace swine (30–40 kg, ~12 weeks).

**Per group:** n=4 (two weeks and four weeks post-induction; control animals).

**Reasoning:** Feasibility for a porcine large-animal platform and alignment with prior swine AAA literature; ethical animal testing following **Charité - 3R principles (Replacement, Reduction, Refinement).**

**Replicates:** Histology/Immunofluorescence/Western Blotting was performed in technical duplicates/multiples per animal; representative slides analysed.

Three consecutive MRI transverse images were analysed and results averaged.

**3. Inclusion and exclusion criteria**

**Inclusion:** clinically healthy female German Landrace, ~12 weeks, 30–40 kg; no complications during AAA induction.

**Exclusion:** Humane endpoints (impending rupture, severe neurological deficit (hindlimb paresis), failed induction.).

**Applied exclusions**: early euthanasia for complications (see above); one non-responder euthanized at study endpoint.

**n for each analysis**: MRI: AAA 2W n=4; AAA 4W n=4; Control n=4. *Ex vivo* (WB/IF/histology/LA-ICP-MS): tissues from the same terminal cohorts (2W n=4, 4W n=4, controls n=4).

IF/WB: A, B, C, E: n=3 each (multiple sections/lanes per animal); D excluded for endpoint analyses.

**4. Randomization**

**AAA-induced animals were randomised to 2W vs 4W MRI follow-up (n=4 each). Controls were imaged post-acclimatisation.**

**5. Blinding**

**During procedures**: Not blinded (operators must know allocation for dosing/device use).

**Outcome assessment**: **MRI images were analysed by three blinded reviewers** measured ROIs and derived CNR/ΔCNR. Immunofluorescence/Western blot quantification used predefined thresholds/normalization.

**Data analysis**: Statistician aware of group labels.

**6. Outcome Measures**

***Primary outcome***

Difference in Change in CNR (ΔCNR) when comparing two-week group, four-week group and control animals.

***Secondary outcomes***

- Aortic diameter expansion by ultrasound.
- ADAMTS4 expression (Western blot densitometry; Immunofluorescence %-area).
- Galectin3 (macrophages) and α-SMA (vascular smooth muscle cells) by WB/IF.
- Gadolinium localisation by LA-ICP-MS.
- Histology (EvG elastin, PSR collagen, Von Kossa calcium) with morphometrics.

**7. Statistical methods**

**Software: IBM SPSS 29.0; GraphPad Prism 10.4.1.**

**Distribution/variance checks: Shapiro-Wilk; Levene’s.**

**Group comparisons: for normal data, one-way ANOVA with Tukey or Dunnett’s T3; for non-normal or unequal variances, Brown-Forsythe/Welch ANOVA with Games-Howell. Two-sided α=0.05; 95% CIs where applicable.**

**Outliers: Grubbs’ test (α=0.05); two values removed before per-animal averaging.**

**8. Experimental animals**

**Species/strain: Sus scrofa domestica, German Landrace.**

**Sex/age/weight: female; ~12 weeks; 30–40 kg.**

**Health status/provenance: clinically examined; vendors: Bundes Hybrid Zuchtprogramm; Gerd Heinrichs.**

**Previous procedures**: None.

**9. Experimental Procedures**

**Where**: Charité – Universitätsmedizin Berlin Research Facilities for Experimental Medicine (FEM); hybrid operating room; clinical 3 T MRI scanner Charité Campus Virchow Klinikum.

**AAA induction (overview): general anaesthesia → infrarenal balloon dilation (14×40 mm; 6–8 bar; 10 min) → occlusion → collagenase 6000 IU + elastase 500 IU (20 min) → CaCl₂ 25%, 0.5 mL (15 min) → angiographic confirmation; Angio-Seal® closure; standard analgesia/antibiotics and ultrasound follow-up.**

**Anaesthesia/monitoring**: Multimodal regimen (atropine, azaperone, ketamine, xylazine, propofol, fentanyl, isoflurane, midazolam); continuous invasive/non-invasive monitoring; fluid/vasoactive and acid–base support as needed.

**Analgesia/antibiotics:** Transdermal fentanyl (50 µg/h), buprenorphine (0.03 mL/kg i.v.), metamizole (i.m. then oral), sulbactam/ampicillin i.v. peri- and post-op.

MRI protocol: axial/sagittal/coronal T1-w; 2D T1 TSE; 3D spoiled GRE; Look-Locker TI selection; 3D IR-GRE late enhancement; pre- and post-ADAMTS4-Gd probe (≈0.03 mmol/kg i.v.).

MRI analysis: three consecutive slices/animal; blinded triplicate ROI measurements for wall, blood, background; ΔCNR computed; identical geometry across time points.

***Ex vivo*: WB (ADAMTS4 ~90 kDa + ~45 kDa processed; Gal-3 ~31 kDa; α-SMA ~42 kDa), IF (ADAMTS4, Gal-3, α-SMA), LA-ICP-MS (Gd mapping), histology (EvG/PSR/Von Kossa).**

**10. Results**

***Primary outcome***

**ΔCNR:** AAA higher than control

- **Two weeks:** 3.18 ± 0.56 **(p = 0.017)**
- **Four weeks:** 4.35 ± 0.84 **(p = 0.046)**
- Control: −0.14 ± 0.57.

***Secondary outcomes***

**Diameter (US):**

- Baseline **0.74 ± 0.10 cm.**
- **Two weeks: 1.59 ± 0.06 cm** (p < 0.001).
- **Four weeks: 1.9 ± 0.19 cm** (p = 0.002).

**ADAMTS4 (*ex vivo*):**

*Western blot*

- **Two weeks: 50.52 ± 3.43 % (p < 0.001).**
- **Four weeks: 90.51 ± 7.44 % (p < 0.001)**.
- Control: **6.22 ± 4.13 %**.

*Immunofluorescence*

- **Two weeks: 43.91 ± 9.48 % (p = 0.006).**
- **Four weeks: 46.97 ± 4.50 % (p < 0.001).**
- Control: **0.39 ± 0.03 %**.

**Inflammation/VSMC:**

*Galectin-3 western blot*

- **Two weeks: 54.12 ± 1.97 % (p < 0.001).**
- **Four weeks: 90.65 ± 7.72 % (p < 0.001).**
- Control: **12.98 ± 3.70 %**.

*α-smooth muscle actin western blot*

- **Two weeks: 33.82 ± 5.54 %** (p < 0.001).
- **Four weeks: 17.28 ± 10.02 %** (p < 0.001).
- Control: **91.43 ± 6.29 %**

**Laser ablation-inductively coupled-mass spectrometry (LA-ICP-MS):**

Focal **Gadolinium** deposition **co-localised** with ADAMTS4-positive regions.

**Histopathology:**

Progressive elastin fragmentation, collagen disorganisation, and calcium deposition in AAA.

***ARRIVE Guidelines: The Recommended Set***

**11. Abstract**

States objectives, model/species, targeted probe, imaging endpoints (ΔCNR), statistical approach, principal findings, and conclusions about early ECM remodelling detection.

**12. Background**

a) **Scientific context**

Rationale for molecular (not size-only) monitoring in AAA; ADAMTS4 as an ECM-remodelling marker; prior murine data; need for large-animal validation.

b) **Relevance of species/model**

Swine cardiovascular anatomy/diameter closely mirror humans; minimally invasive approach improves translational fit for endovascular therapies and adheres to the **Charité - 3R principles (Replacement, Reduction, Refinement).**

**13. Objectives**

**Research question**

Can ADAMTS4-targeted molecular MRI detect ECM remodelling in porcine AAA before marked diameter enlargement and align with ex-vivo markers?

**Hypotheses** (pre-specified)

1. **ΔCNR** after ADAMTS4-Gd is **greater** in AAA vs controls at 2W/4W.
2. **ΔCNR** correlates with **ADAMTS4** expression (IF/WB) and focal **Gadolinium** by LA-ICP-MS.
3. Imaging changes **precede** significant diameter growth, providing activity information beyond size.
4. The protocol is **feasible** in swine and possible in clinical 3 T MRI.

**14. Ethical Statement**

**All procedures were approved by the State Office for Health and Social Affairs Berlin (LaGeSo) and conducted in accordance with FELASA and ARRIVE guidelines and the Charité - 3R principles (Replacement, Reduction, Refinement).**

**15. Housing and Husbandry**

**Pigs were housed in groups of three per pen (12.86 m^2^; tiled; straw/sawdust bedding) throughout acclimation and the study. Enrichment (chains, balls, rubber toys) was available continuously. Natural daylight plus artificial lighting 06:00 - 18:00; temperature 20 ± 2 °C, humidity 55 ± 10 %. Water ad libitum via self-drinkers; Ssniff pellets 70 g/kg/day in two meals with hay; food rewards used during training. Pens were cleaned daily with replacement of leftovers and bedding. Where possible, presumed sibling animals were housed together, but siblings were not always available/confirmed.**

**16. Animal Care and Monitoring**

a) **Refinements to reduce pain/distress**

Endovascular (minimally invasive) induction; multimodal anaesthesia; mechanical ventilation; peri-/post-op analgesia and antibiotics; vascular closure devices; habituation for awake US follow-ups.

b) **Monitoring**

**Continuous intra-op monitoring; scheduled US weekly; MR under GA with ventilation.**

c) **Humane endpoints & monitoring frequency**

Humane endpoints included clinical signs of rupture/severe distress; intra-op continuous monitoring; twice daily clinical checks for 14 days post-op, following once daily; euthanasia under deep anaesthesia when endpoints were met; no adverse reactions to ADAMTS4 probe reported.

**17. Interpretation/Scientific Implications**

a) **Interpretation**

ADAMTS4-targeted MRI detects active ECM remodelling in vivo and aligns with ex-vivo ADAMTS4/Gd localisation, offering molecular activity readouts beyond size for potential risk stratification and therapy monitoring.

b) **Limitations**

Small n(=4/group); female-only, young animals (~12 weeks); high drop-out rate in early experimental phase.

**18. Generalizability/Translation**

**Use of swine (human-like aortic size/ECM) and clinical 3 T MRI supports translation. The targeted approach complements molecular MRI probes currently in clinical application.**

**19. Protocol Registration**

**A pre-specified experimental protocol (research question, design, and analysis plan) was prepared before the study and registered/approved with LaGeSo under G 77/22 (State Office for Health and Social Affairs Berlin).**

**20. Data Access**

Original data generated during this study are available upon reasonable request.

**21. Declaration of Interests**

Authors declare **no competing interests.**

| Correlation | Spearman r | p-values | Confidence Interval of rs |
| --- | --- | --- | --- |
| ∆CNR vs. ADAMTS4 | 0.9161 | 0.00008 | 0.7121 to 0.9774 |
| ∆CNR vs. Gal-3 | 0.8741 | 0.0004 | 0.5900 to 0.9656 |
| ∆CNR vs. α-SMA | –0.9021 | 0.0002 | –0.9735 to –0.6700 |
| ADAMTS4 vs. Gal-3 | 0.8462 | 0.0009 | 0.5152 to 0.9575 |
| ADAMTS4 vs. α-SMA | –0.8531 | 0.0007 | –0.9596 to –0.5335 |
| Gal-3. vs. α-SMA | –0.8741 | 0.0004 | –0.9656 to –0.5900 |

**Supplementary Tables**

**Table S-I**

Table S-I. **Spearman correlation analysis between MRI contrast and extracellular matrix remodeling markers.**

Pairwise Spearman rank correlations (rs) were calculated to assess relationships between in vivo MRI-derived change in contrast-to-noise ratio (∆CNR) and molecular markers of extracellular matrix remodeling in aneurysmal aortic tissue. Positive values indicate direct associations, while negative values indicate inverse relationships. Corresponding p-values and 95% confidence intervals for each correlation coefficient are reported.

**Table S-II**

| Parameter | Value |
| --- | --- |
| Number of observations | 12 |
| Model | Simple linear regression (least squares) |
| *F* statistic | *F*(1,10) = 23.84 |
| *p* value | 0.0006 |
| *R*^2^ | 0.7045 |
| Intercept (*β*_0_) | −0.1172 (95% CI −1.560 to 1.325; *p* = 0.8600) |
| ADAMTS4 coefficient (*β*_1_) | 1.805 × 10^−6^ (95% CI 9.815 × 10^−7^ to 2.629 × 10^−6^; *p* = 0.0006) |
| Residual normality (Shapiro-Wilk) | *p* = 0.3317 |

Table S-II. **Simple linear regression analysis of ΔCNR versus ADAMTS4-positive cells.**

Linear regression analysis demonstrating a significant positive association between ΔCNR and ADAMTS4-positive cell area. Approximately 70% of the variance in ΔCNR is explained by ADAMTS4 expression. Residual diagnostics confirmed normal distribution of residuals; *N* = 12.

**Supplementary Figures**


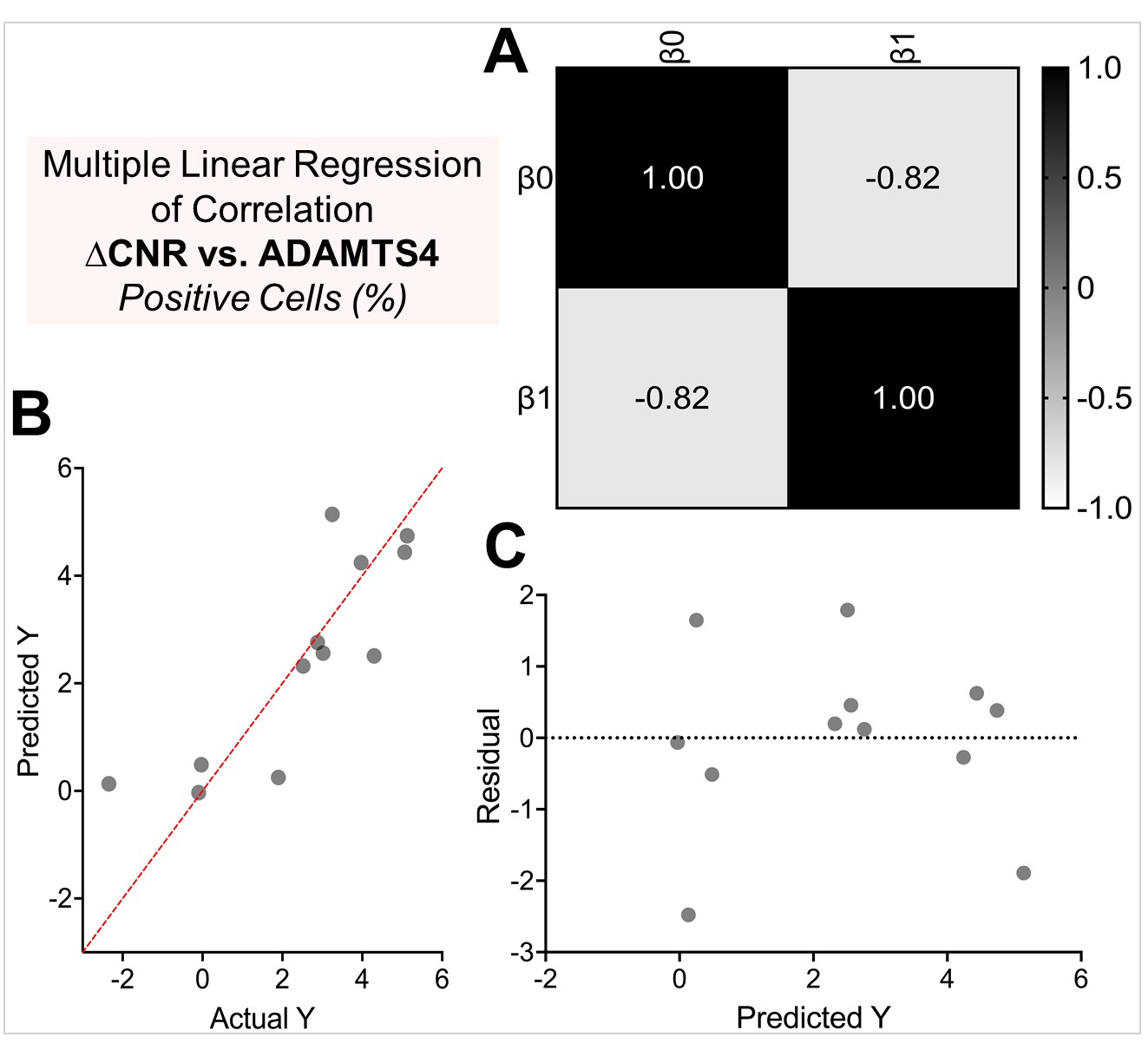
**Figure S-I**

Fig. S-I. **Linear regression analysis of ΔCNR versus ADAMTS4-positive cells.**

**A**, Correlation matrix of model coefficients (*β*_0_, *β*_1_) illustrating parameter relationships in the linear regression model. **B**, Scatter plot of measured ΔCNR values versus model-predicted ΔCNR values with least-squares regression line, demonstrating strong agreement between observed and fitted data. **C**, Residual plot showing homoscedastic distribution of residuals around zero without systematic deviation. Simple linear regression revealed a strong positive association between ΔCNR and ADAMTS4-positive area (*R*^2^ = 0.7045, *F*(1,10) = 23.84, *p* = 0.0006). Residuals passed normality testing (Shapiro-Wilk *p* = 0.3317), supporting model validity; *N* = 12.


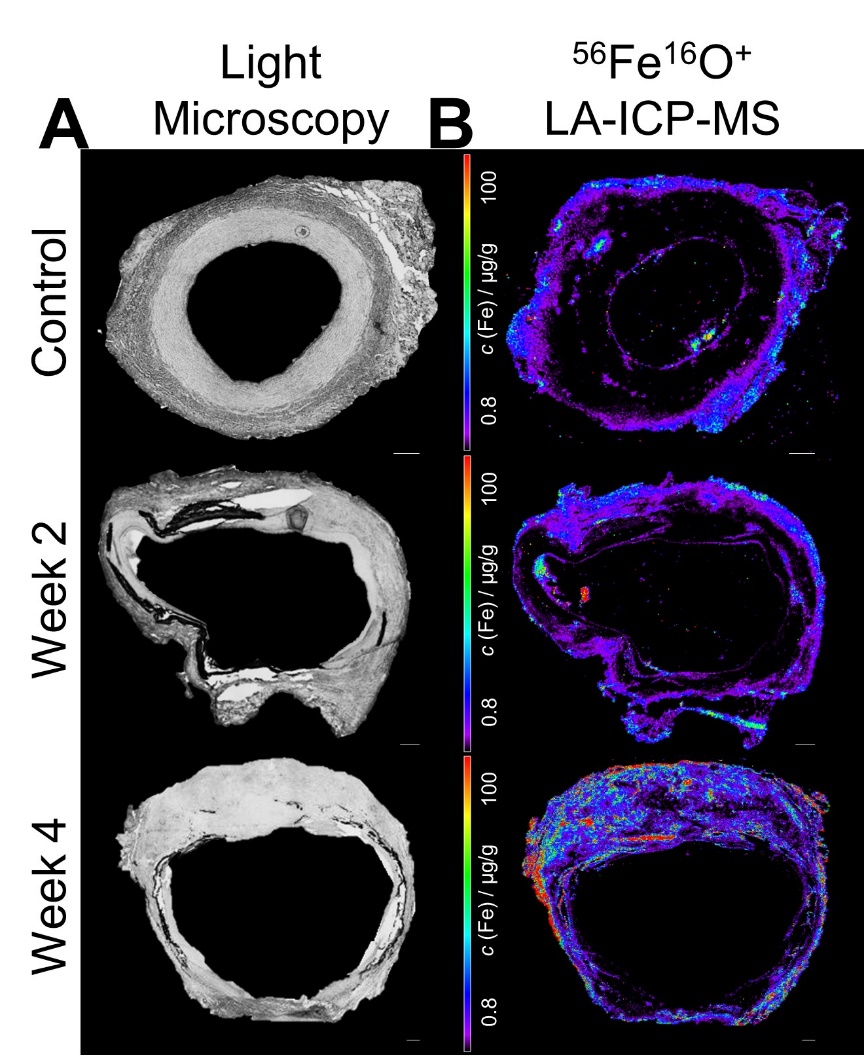
**Figure S-II**

Fig. S-II. **^56^Fe^16^O^+^ laser ablation-inductively coupled plasma-mass spectrometry mapping of infrarenal porcine aortic tissue.**

**A**, Representative bright-field light microscopy cross-sections of infrarenal aorta from control animals, two weeks (2W) after abdominal aortic aneurysm induction, and four weeks (4W) post-intervention. **B**, Corresponding ^56^Fe^16^O^+^ laser ablation-inductively coupled plasma-mass spectrometry elemental maps illustrating spatial iron distribution within the vessel wall. Color scale represents relative iron signal intensity (counts per second, cps). Control tissue demonstrates homogeneous low-level iron distribution. At 2W, focal iron signal increases are observed, while 4W specimens show more pronounced and heterogeneous iron accumulation within the remodeled aneurysmal wall. Scale bars: 500 µm.

**Supplementary Necropsy Images**

***Euthanasia at Two Weeks Post-Intervention (n=4)***


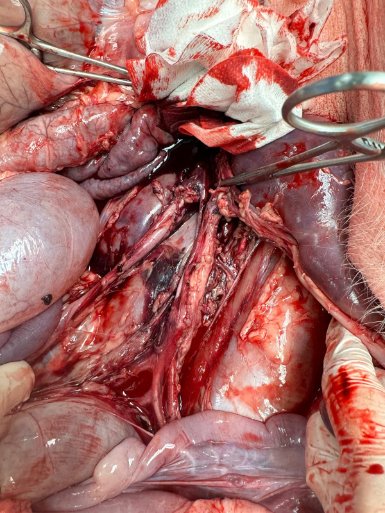

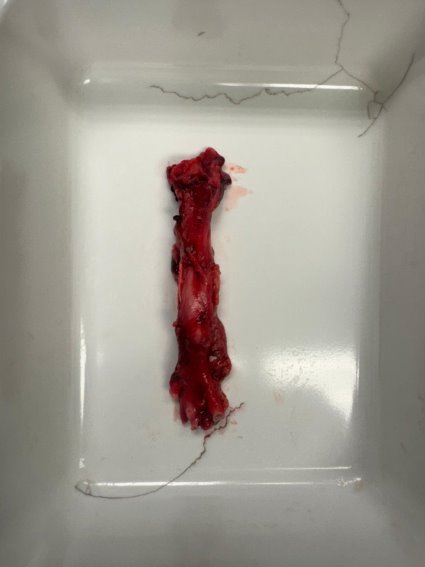

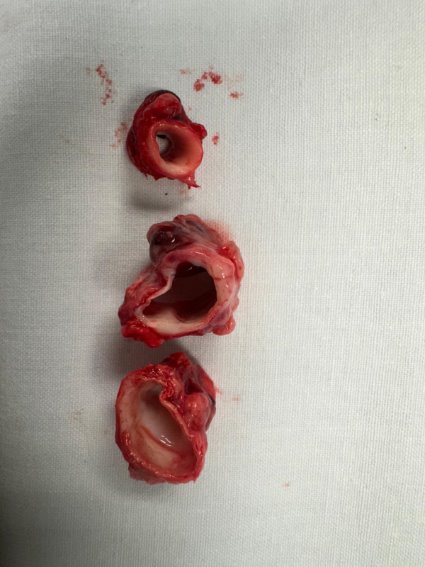
**Animal 1**


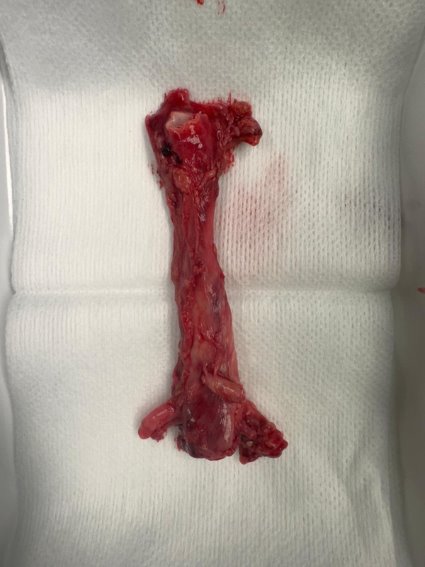

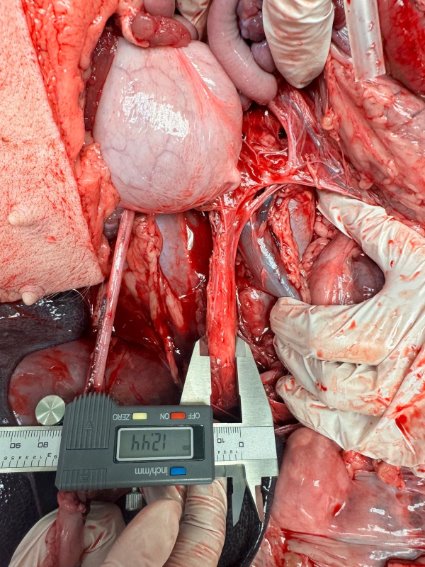
**Animal 2**

**
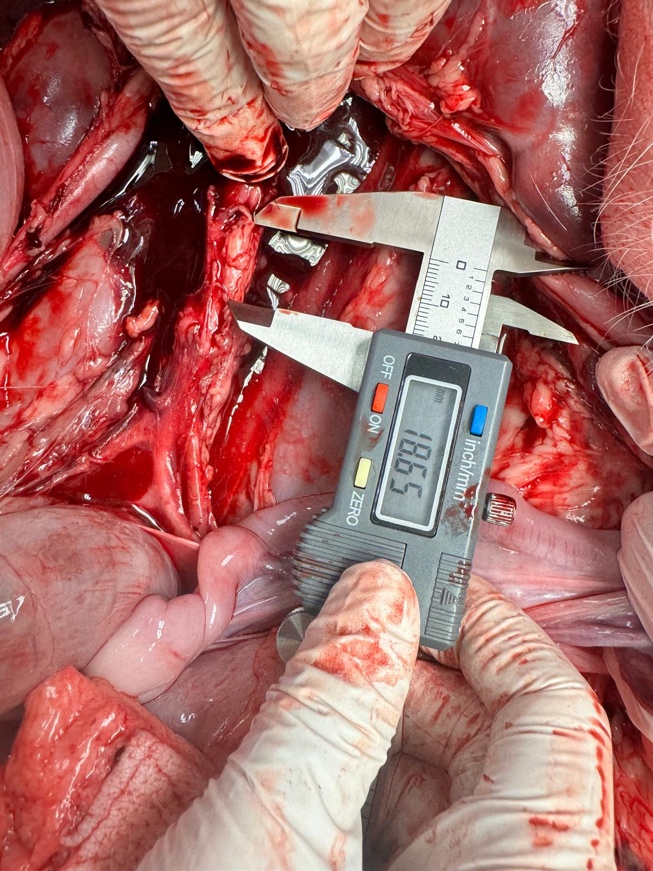

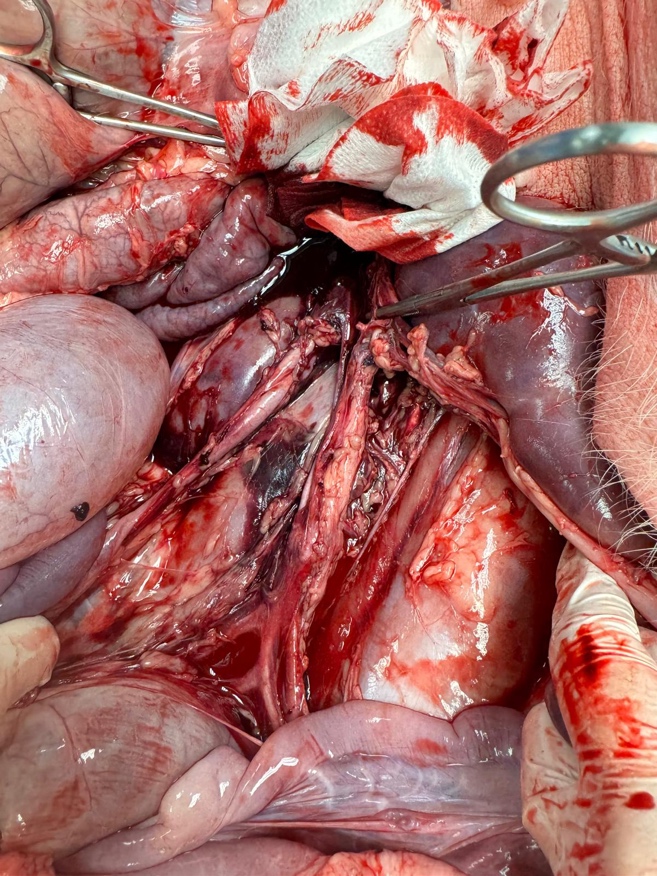
Animal 3**

**
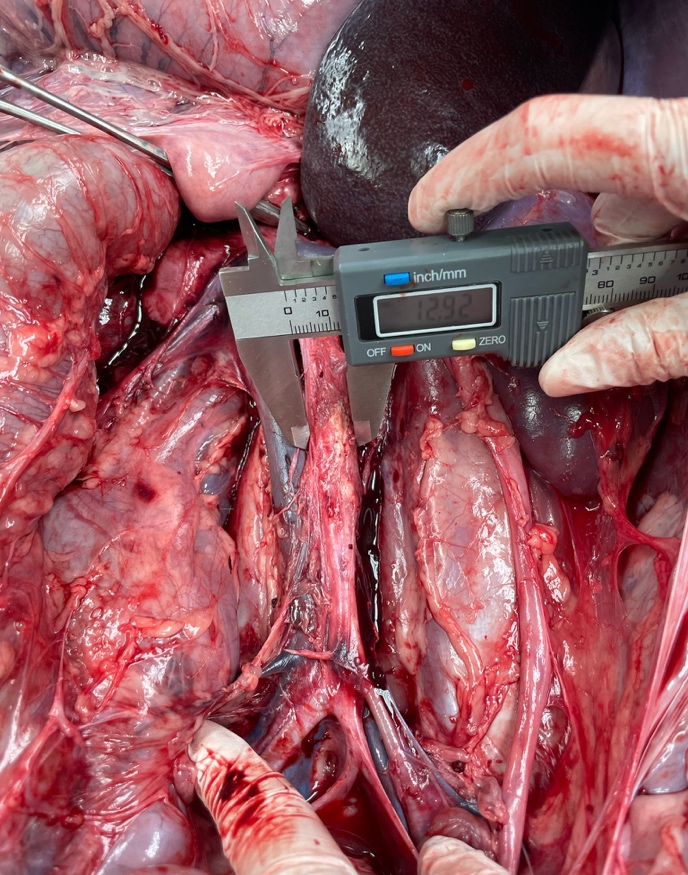

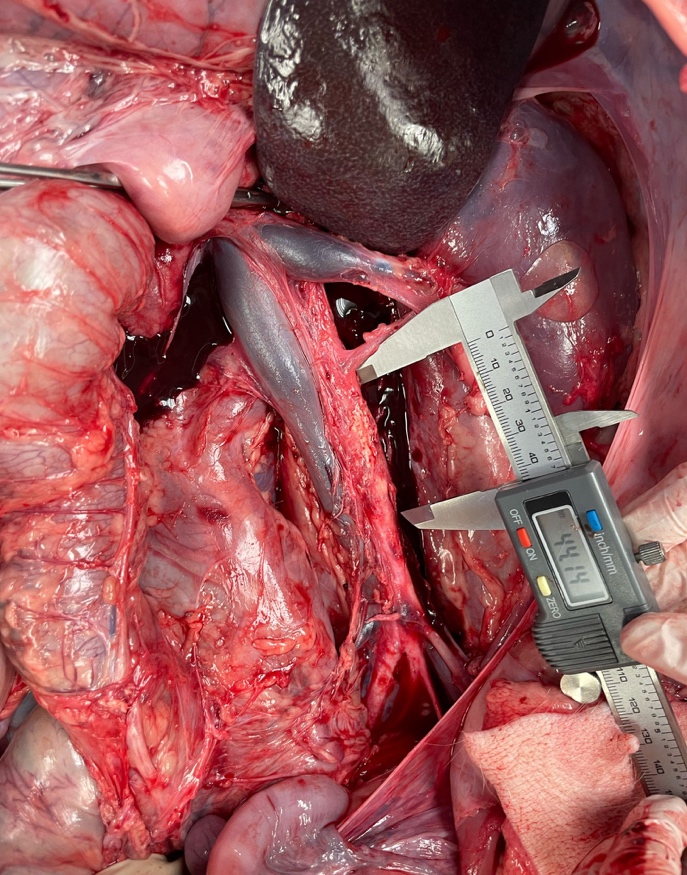
Animal 4**

***Euthanasia at Four Weeks Post-Intervention (n=4)***


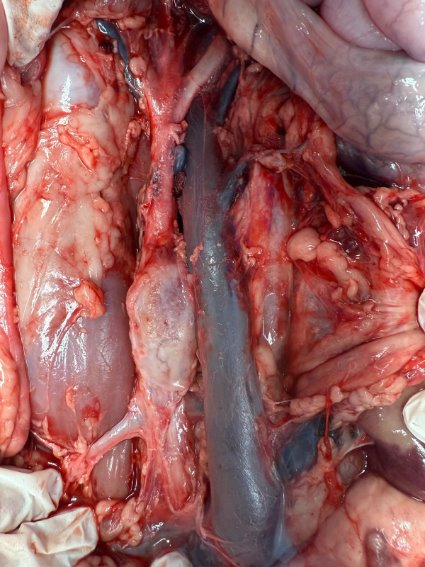

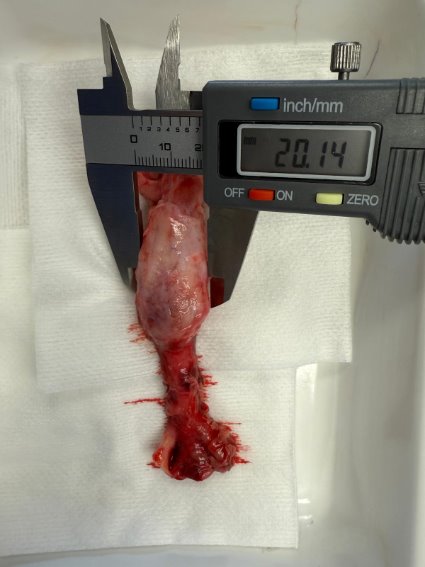

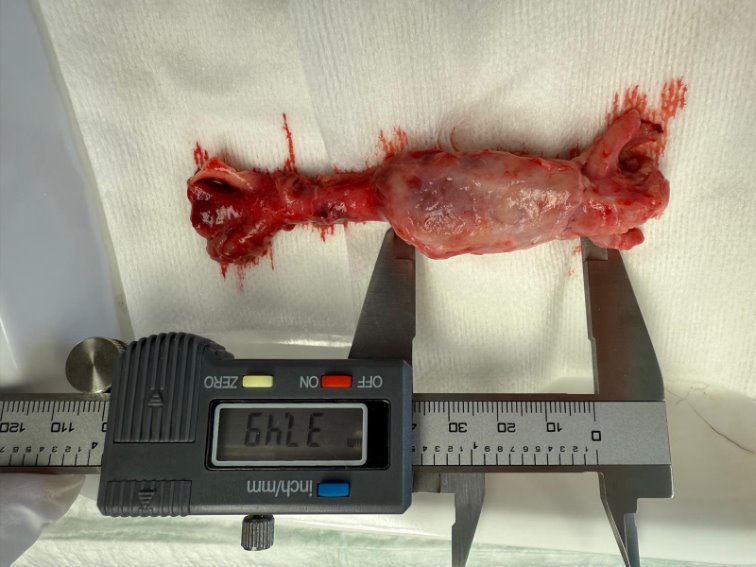
**Animal 5**


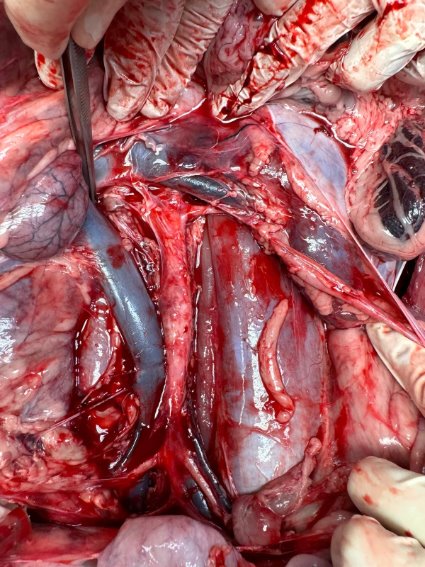

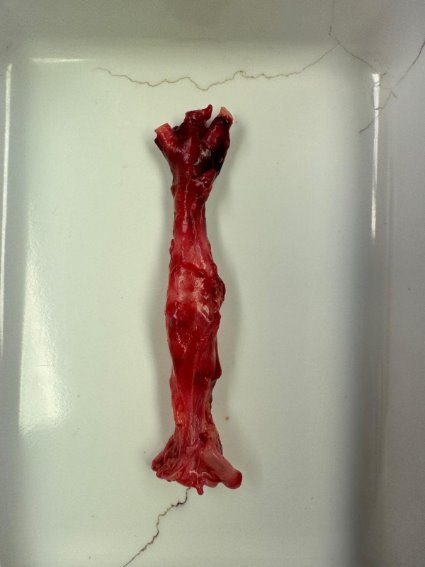
**Animal 6**

**
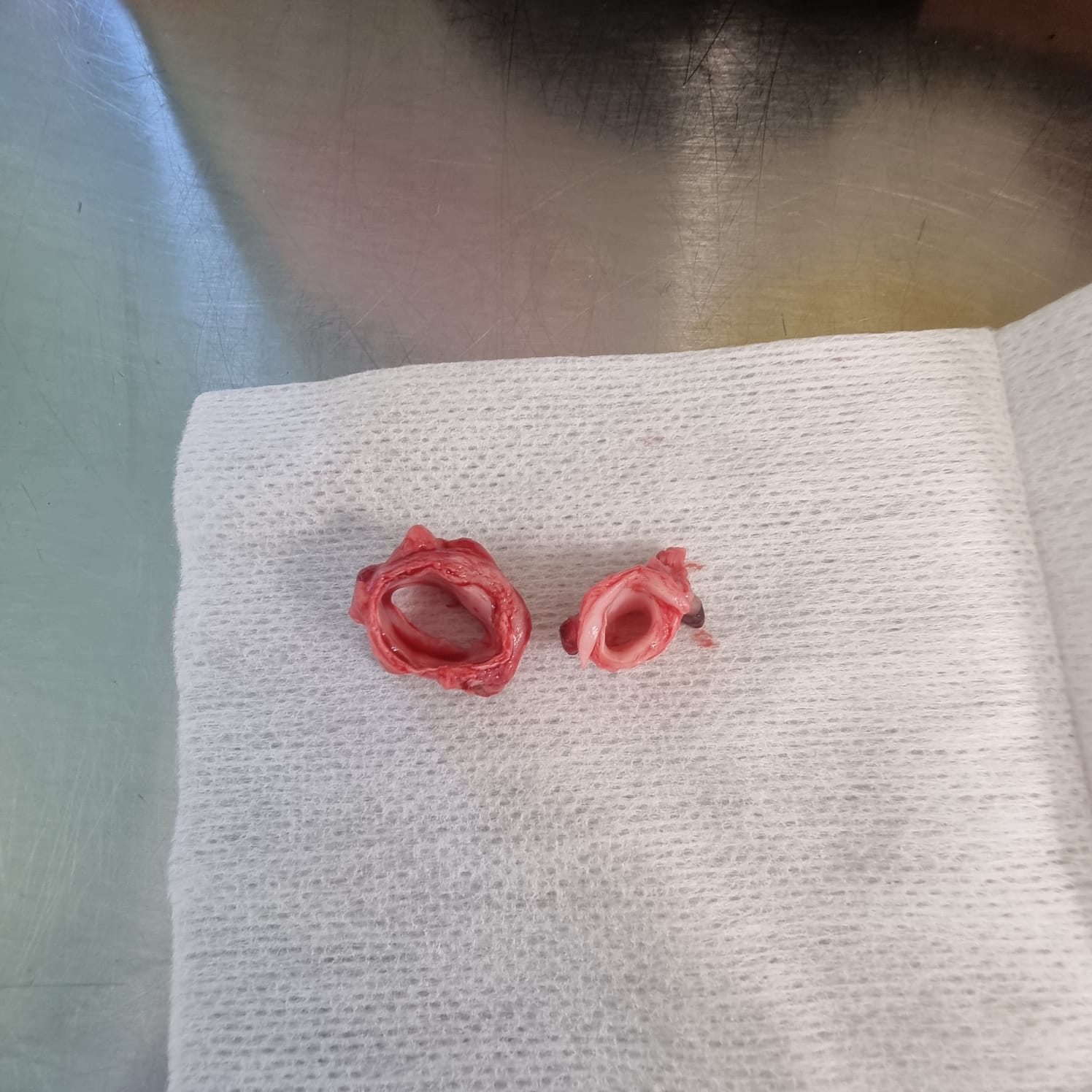

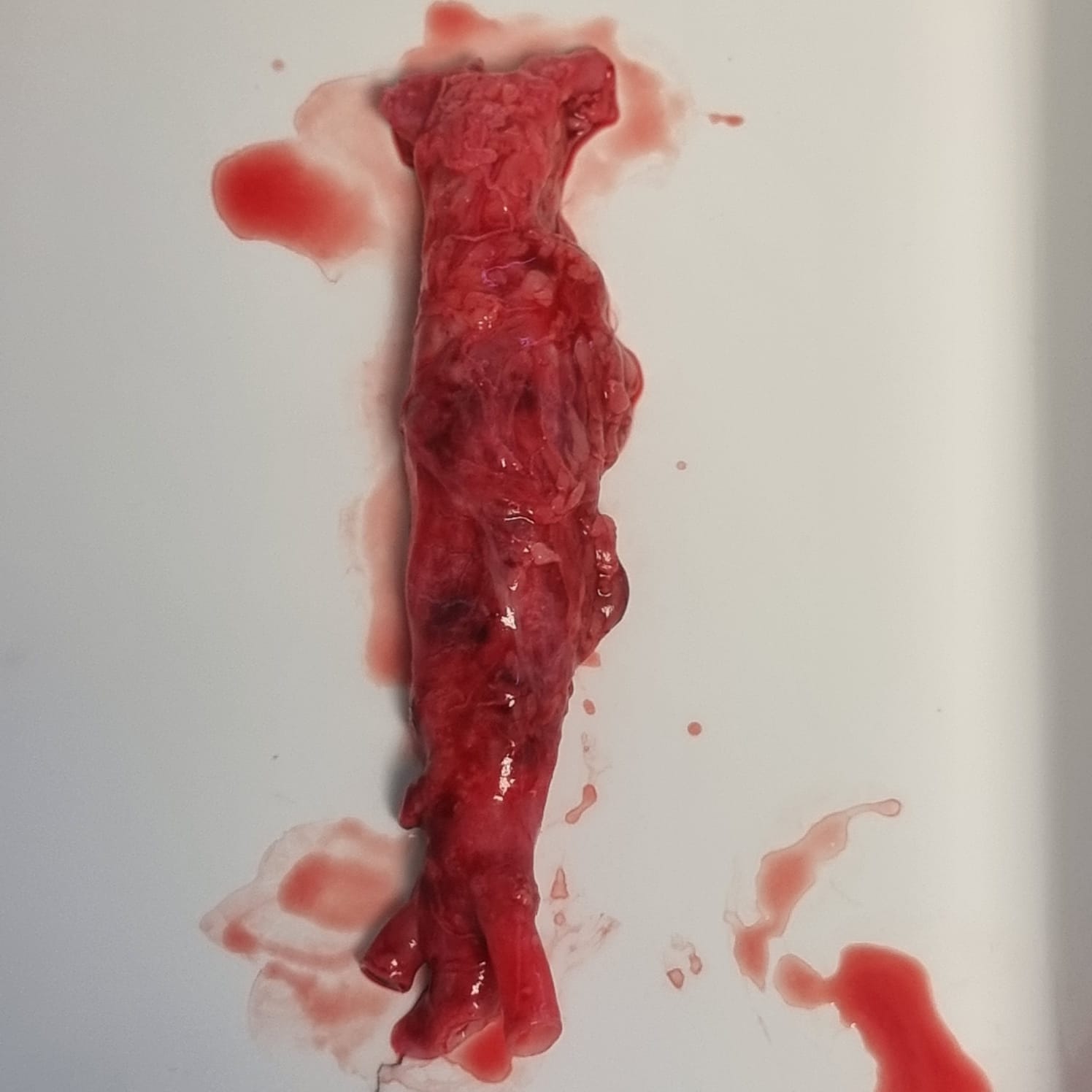

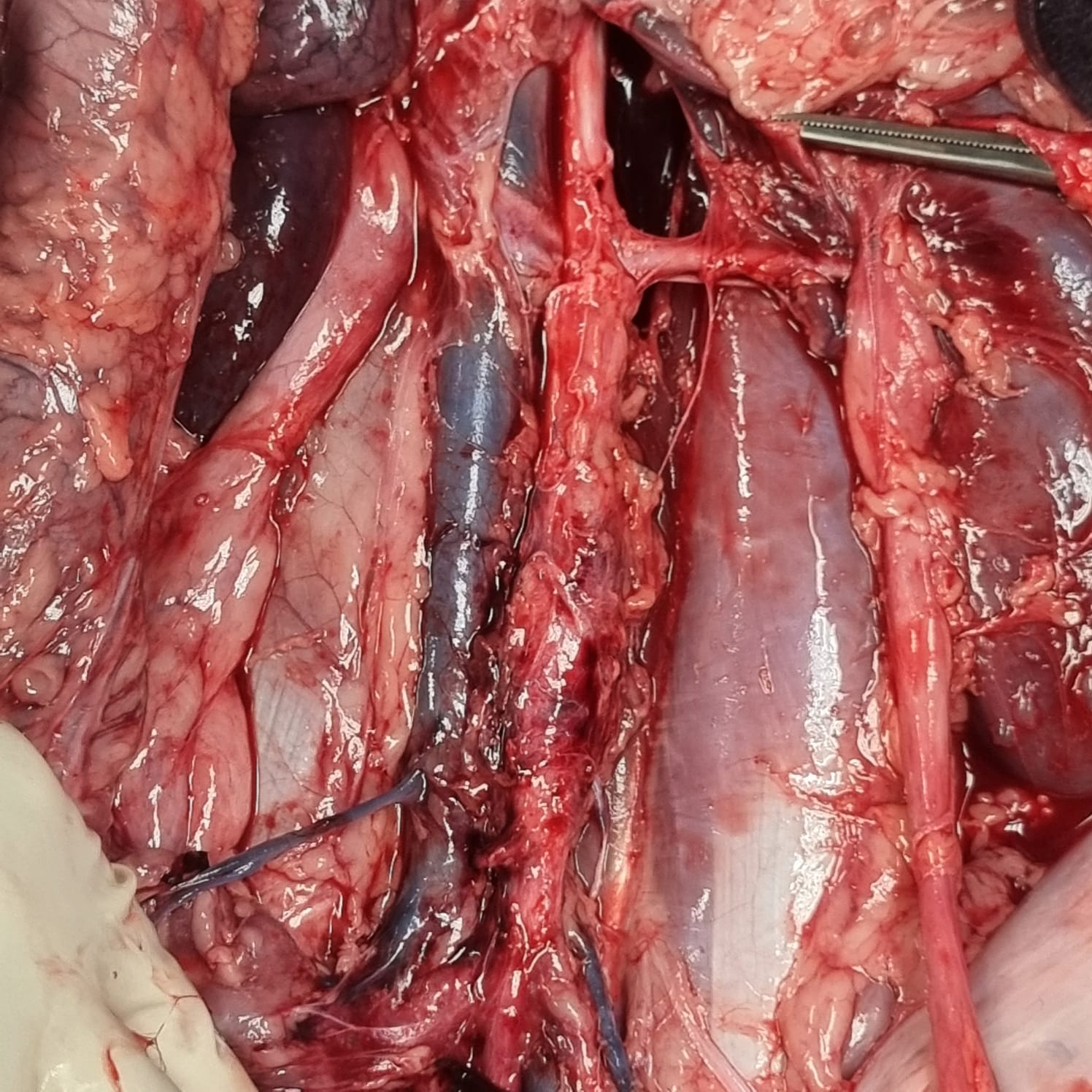
 Animal 7**

**
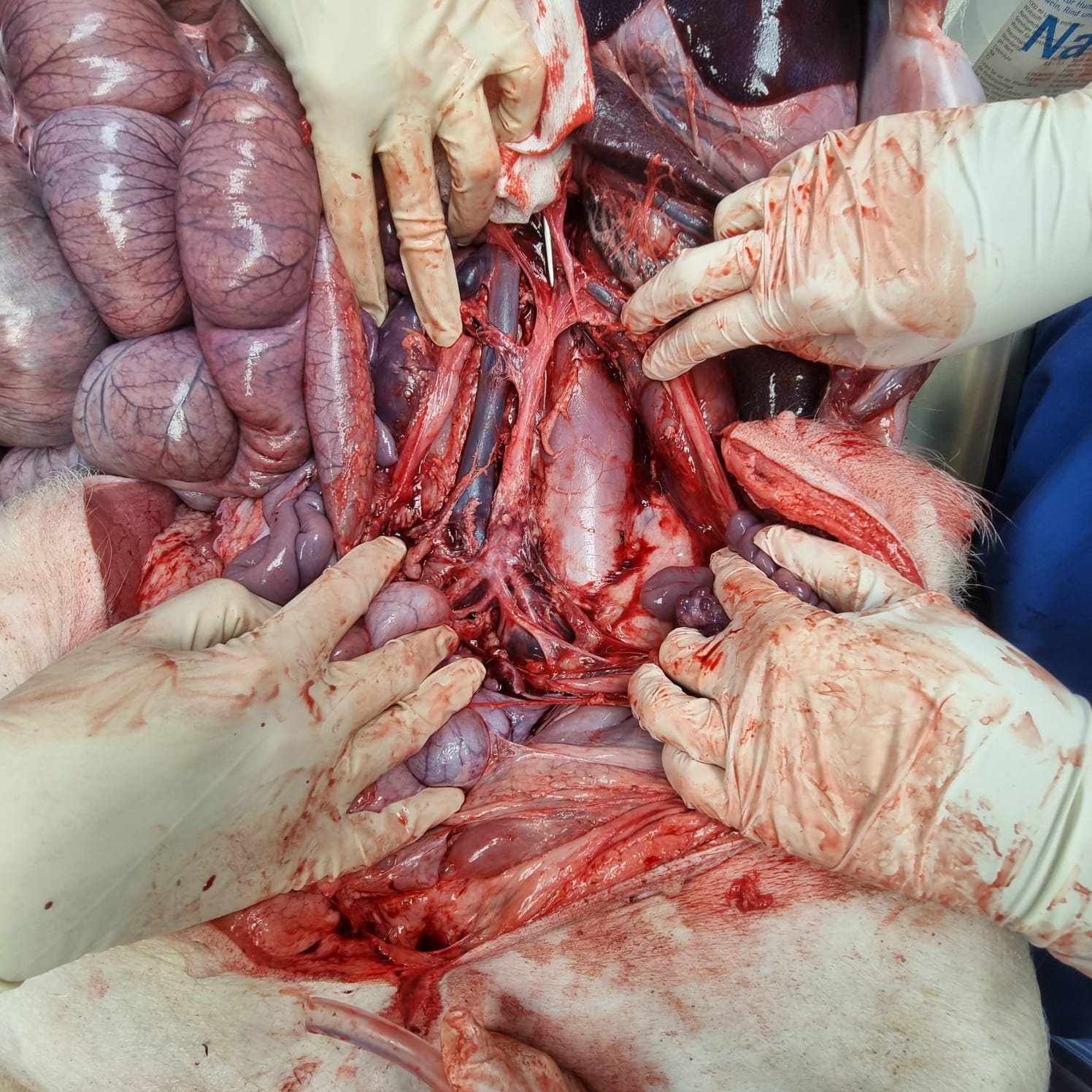

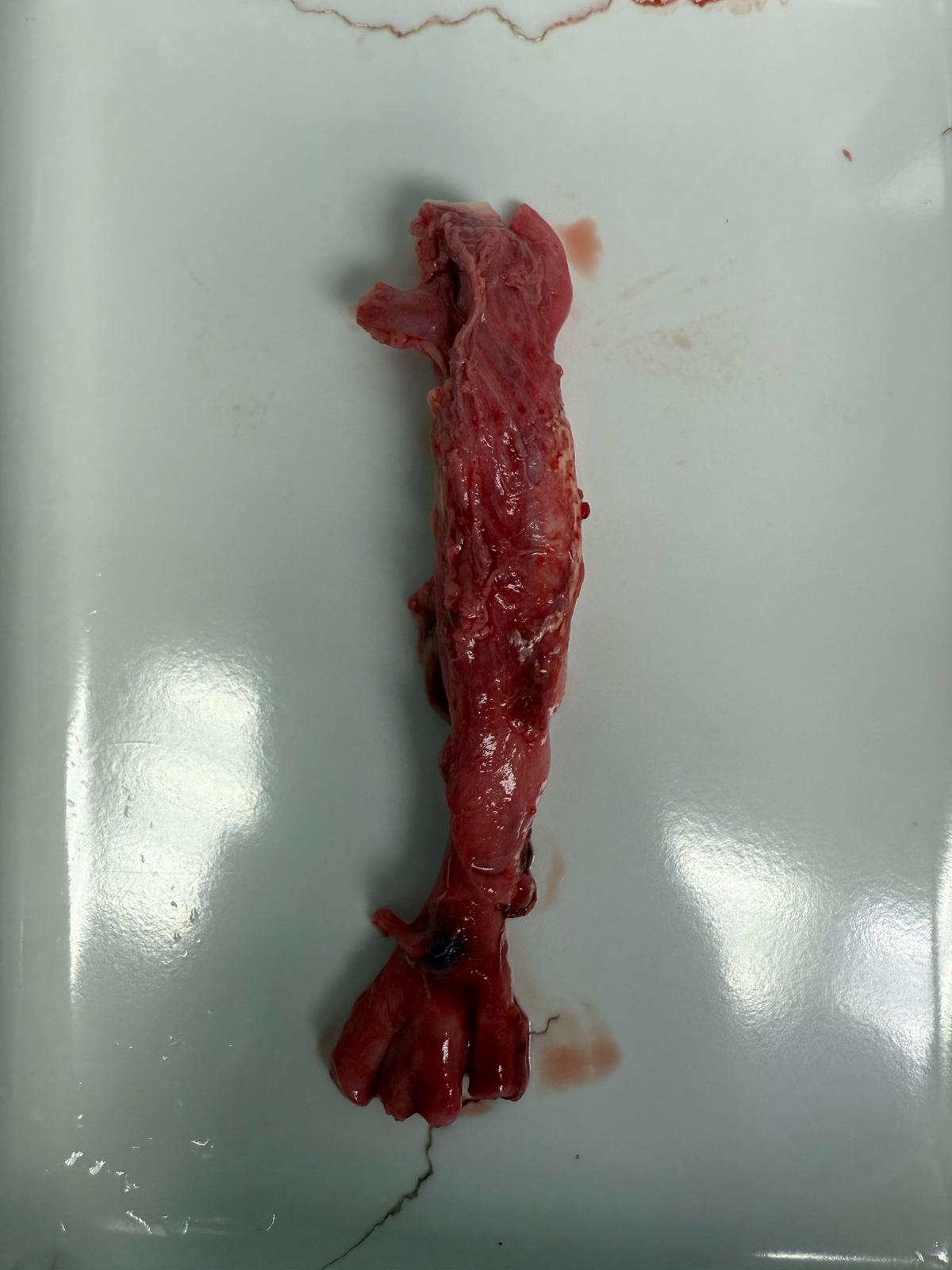

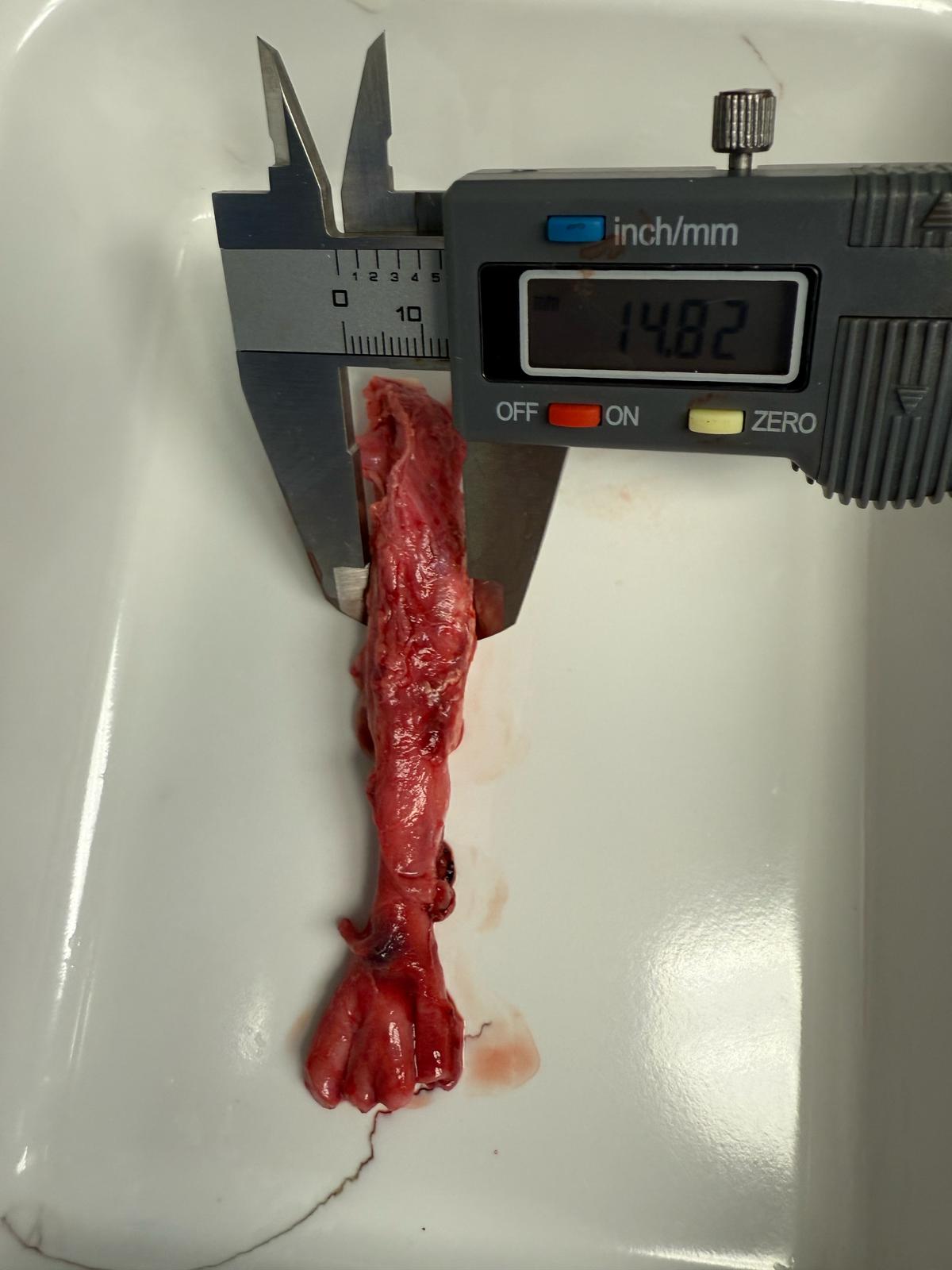
Animal 8**

***Healthy Control Animals (n=4)***

**
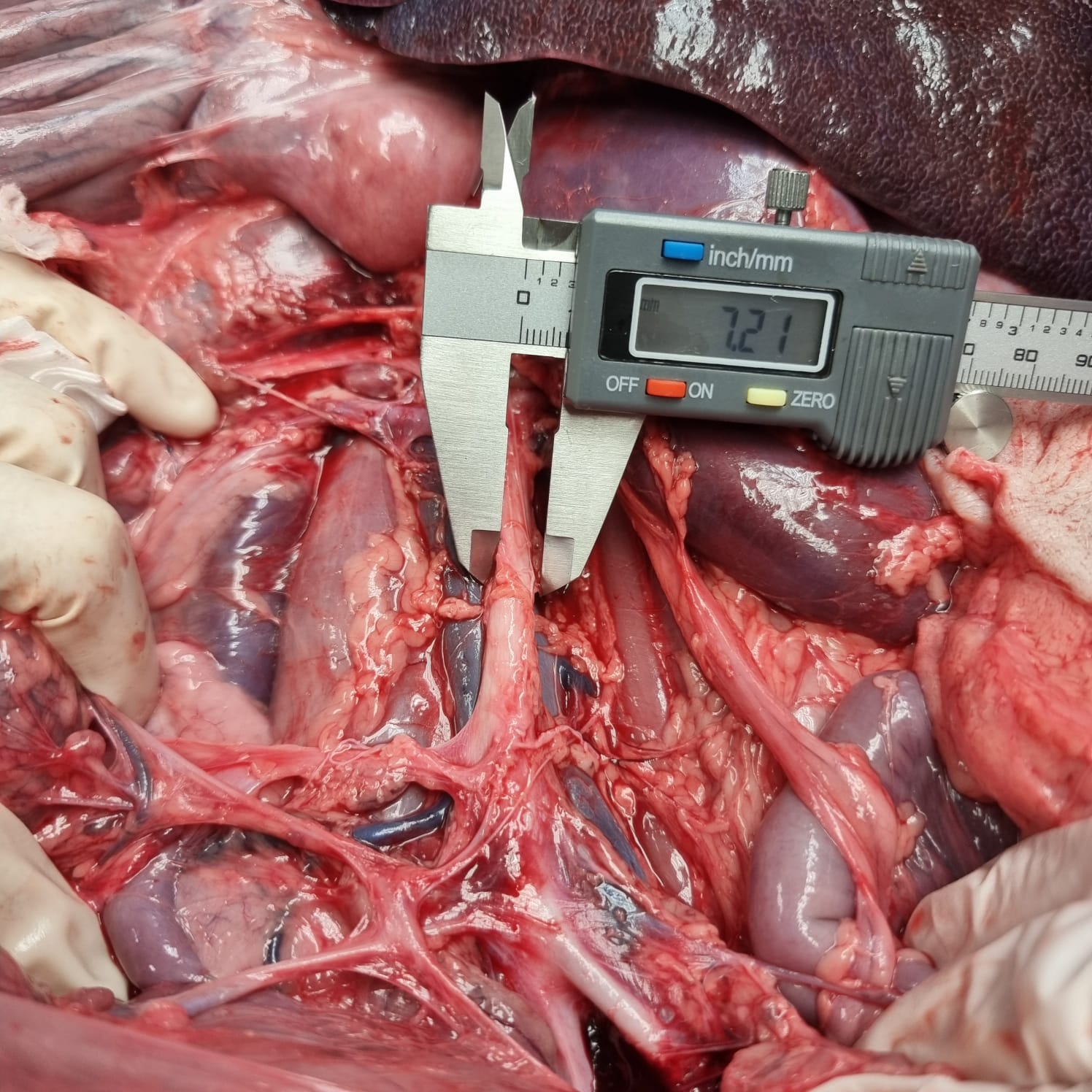

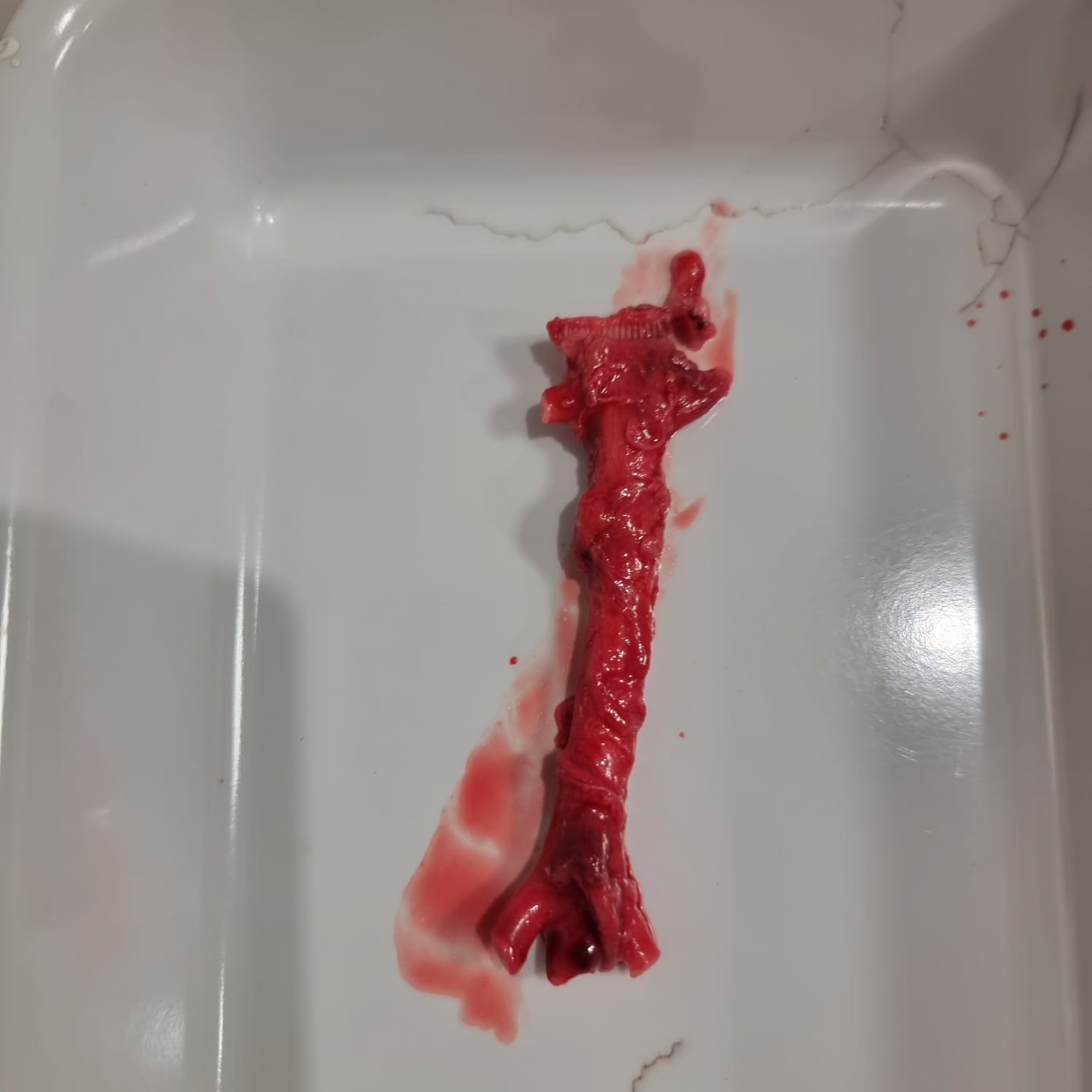
Control Animal 1**

**
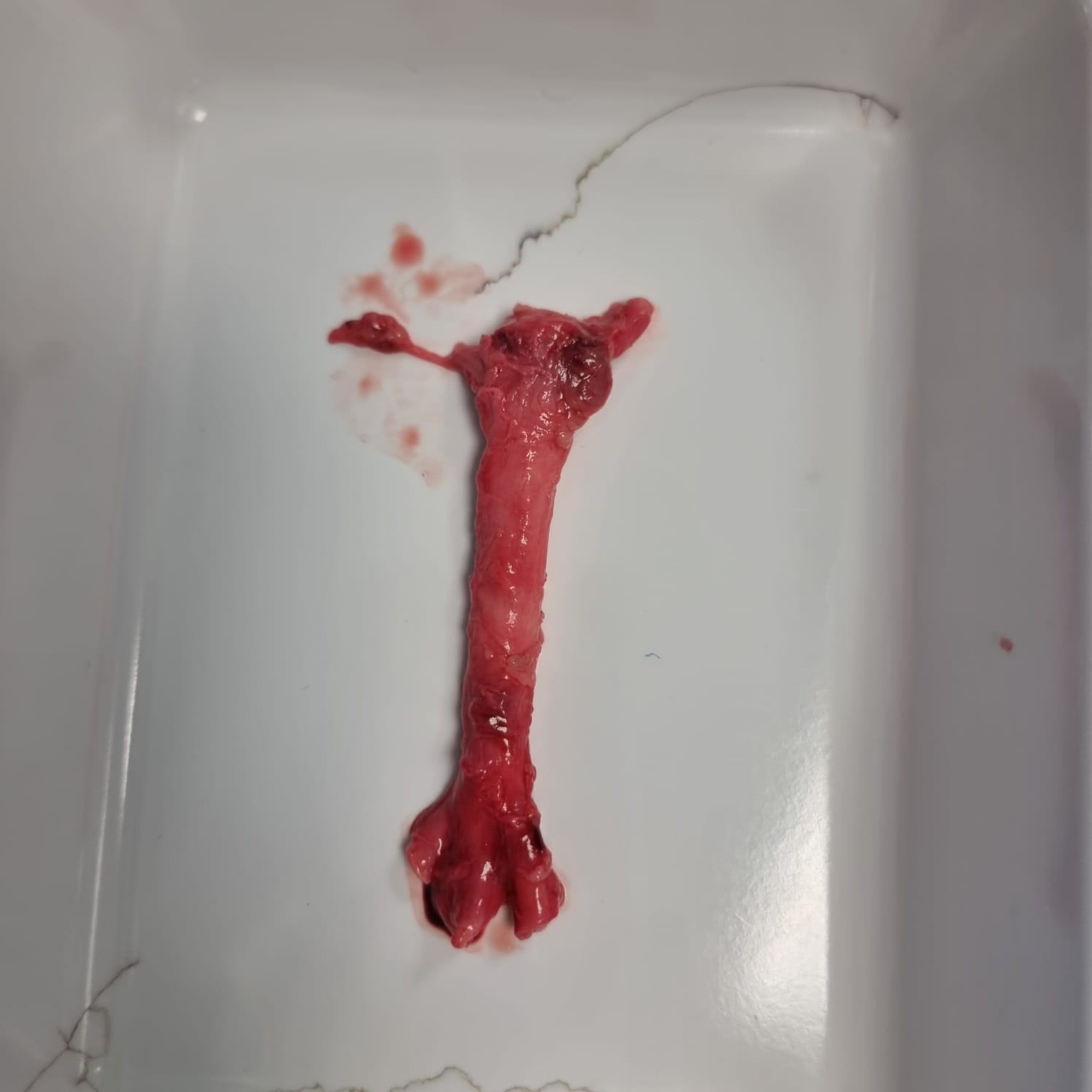

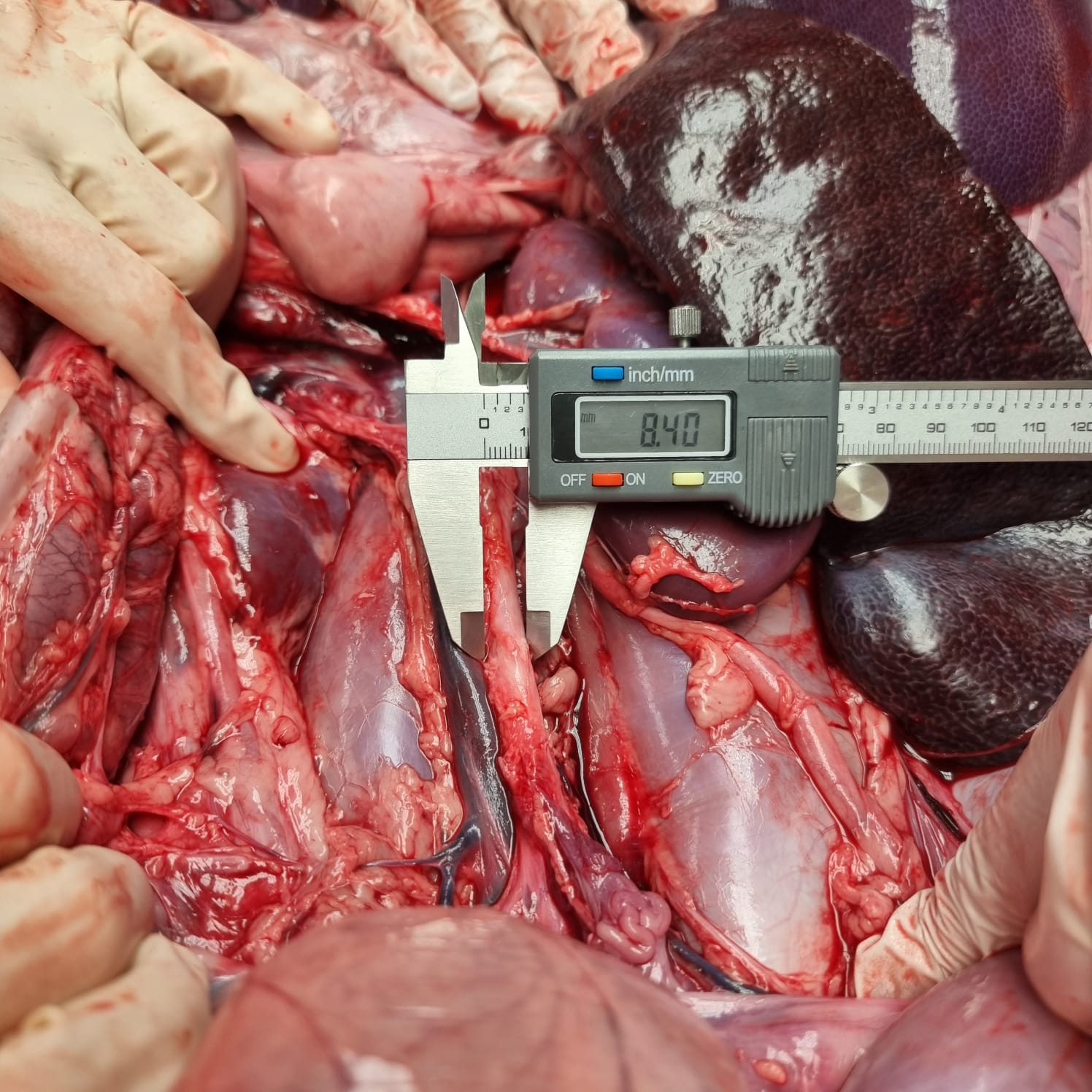
Control Animal 2**

**
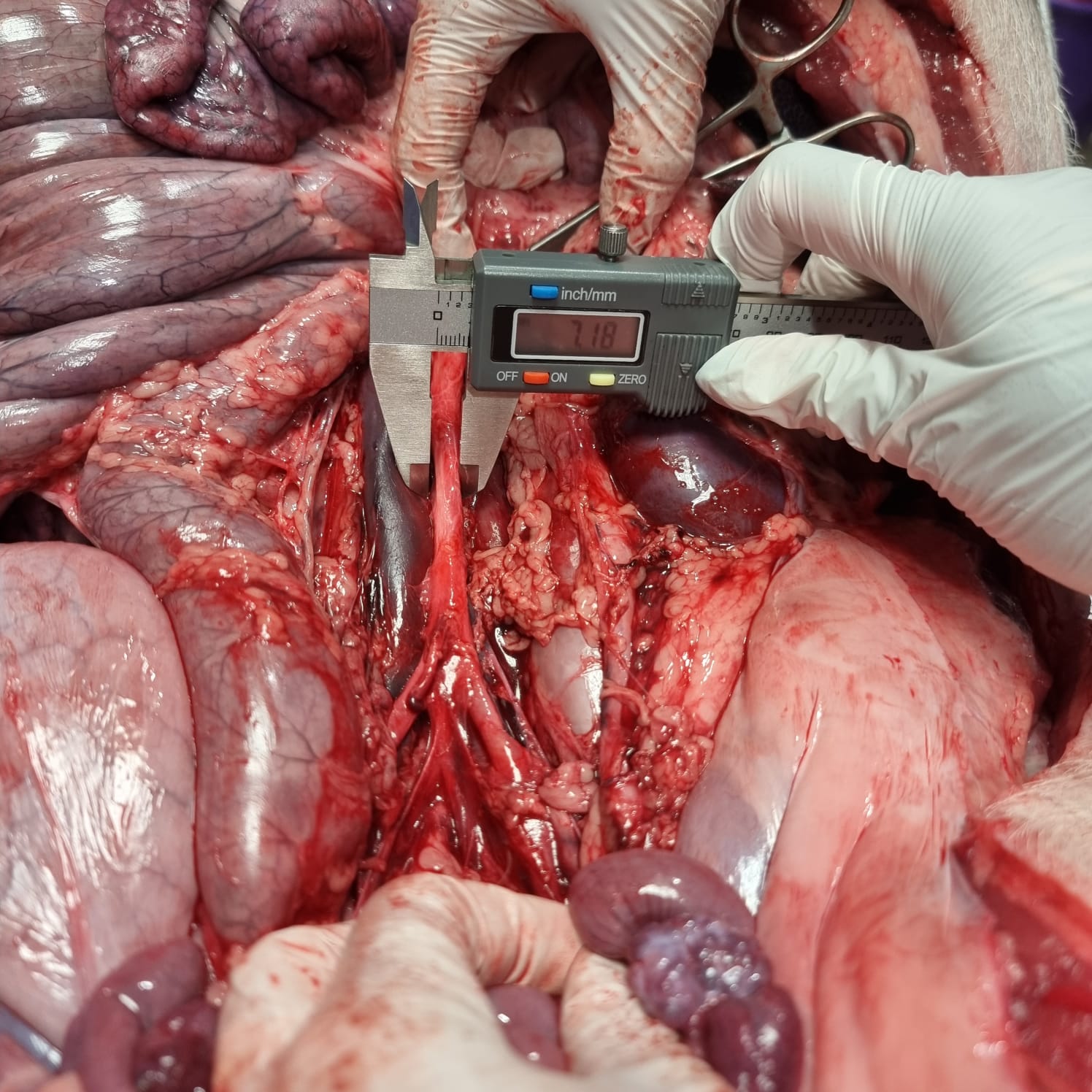

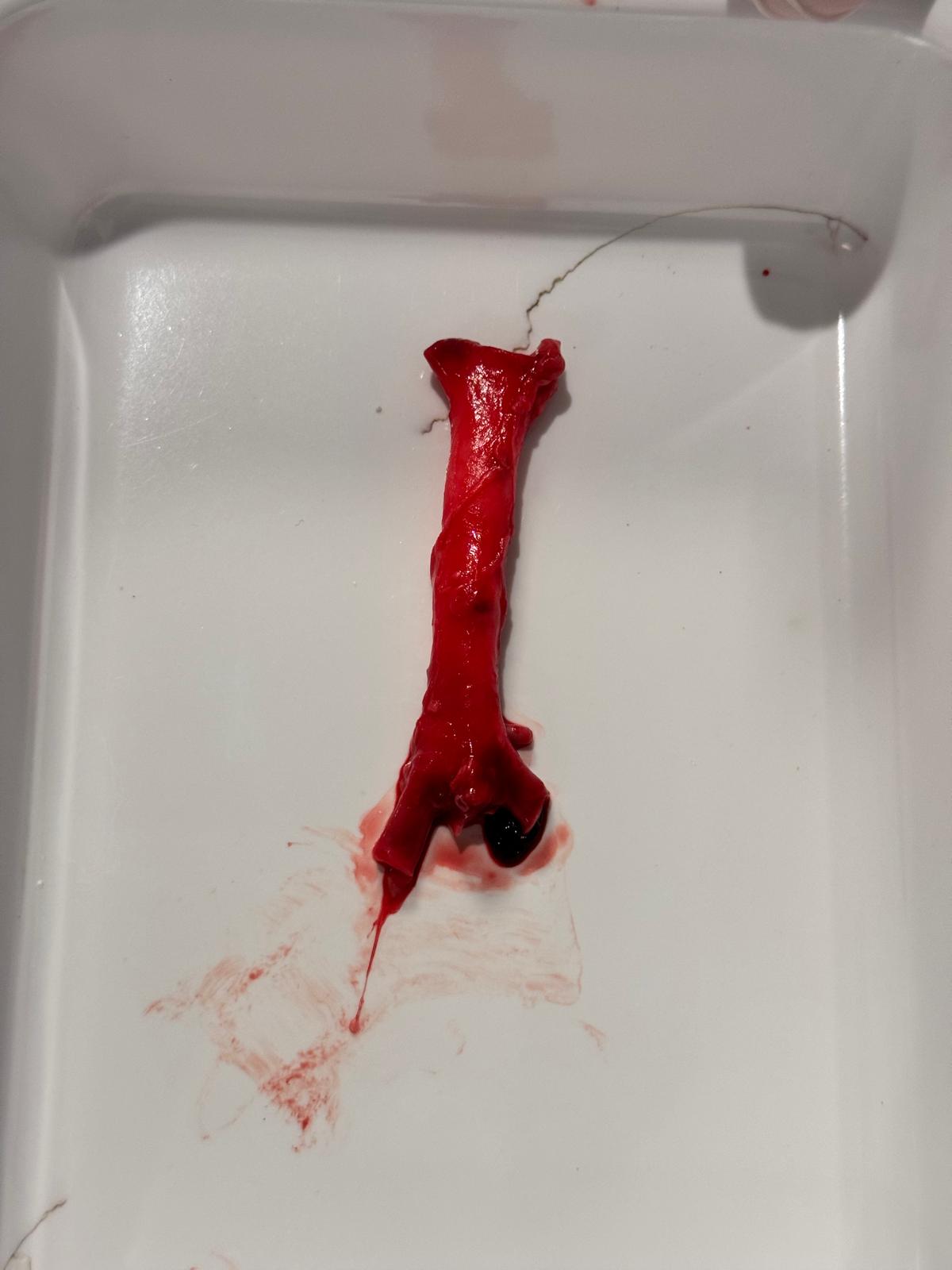
Control Animal 3**

**
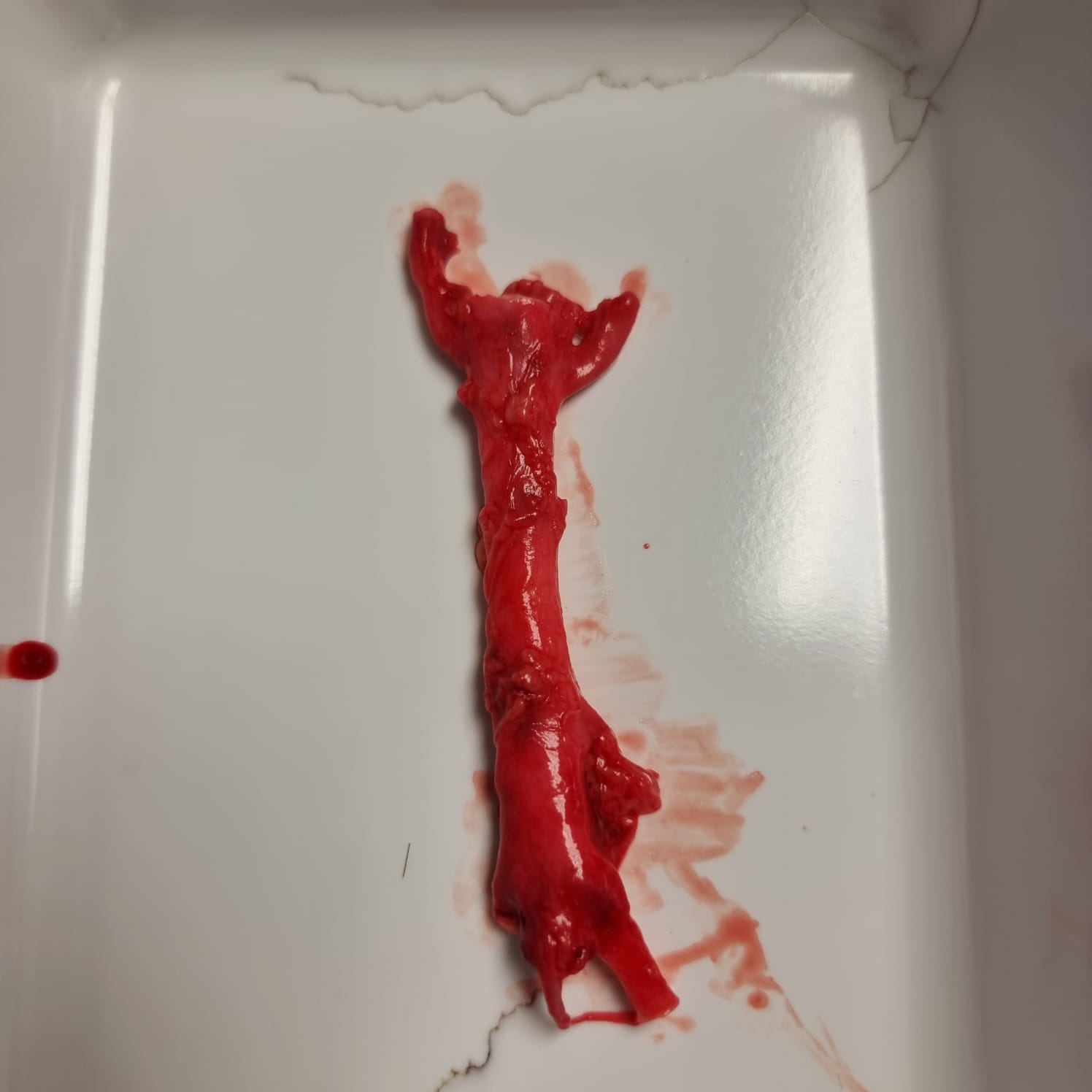

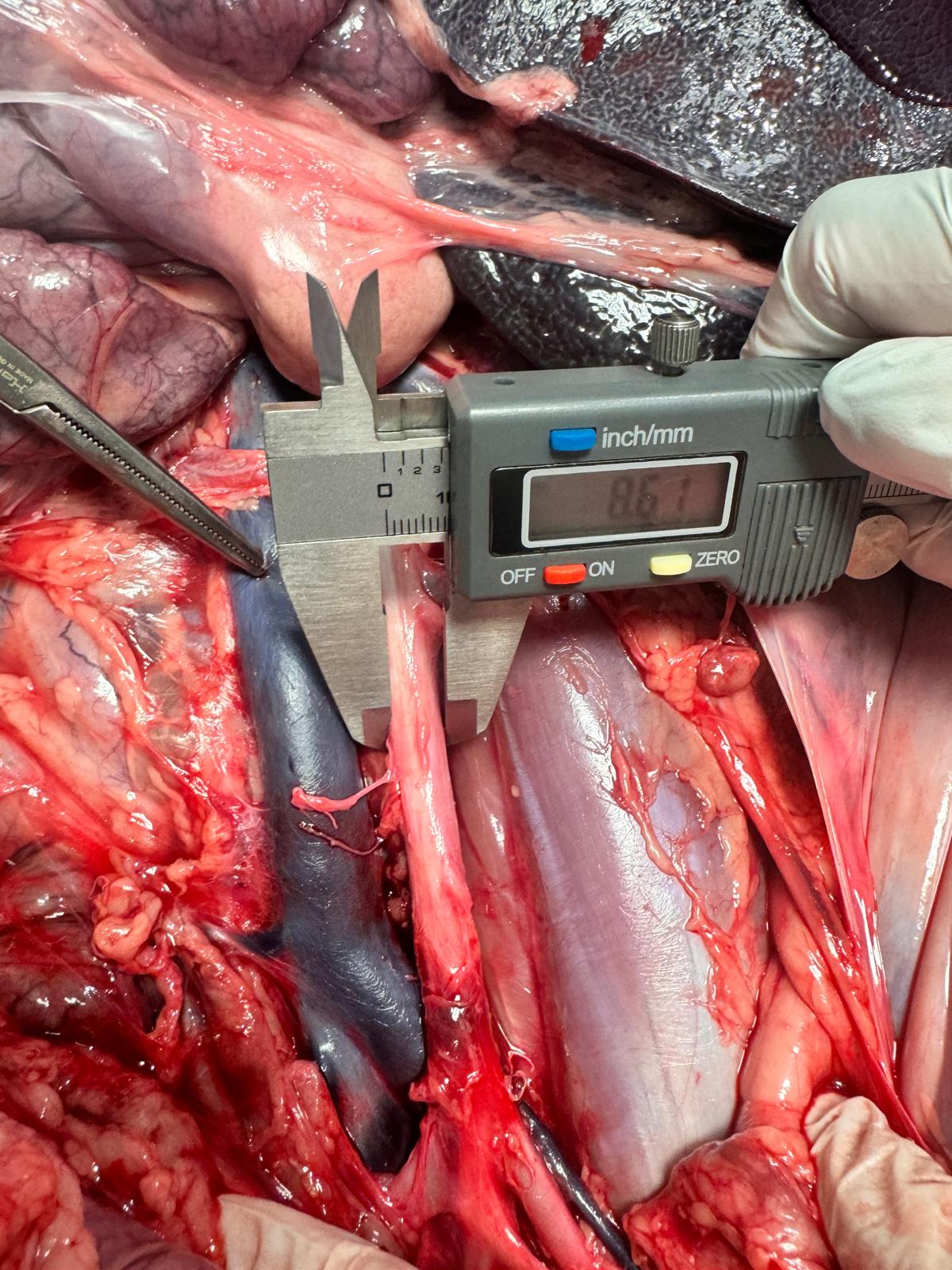
Control Animal 4**

**Supplementary Figure Material**

*
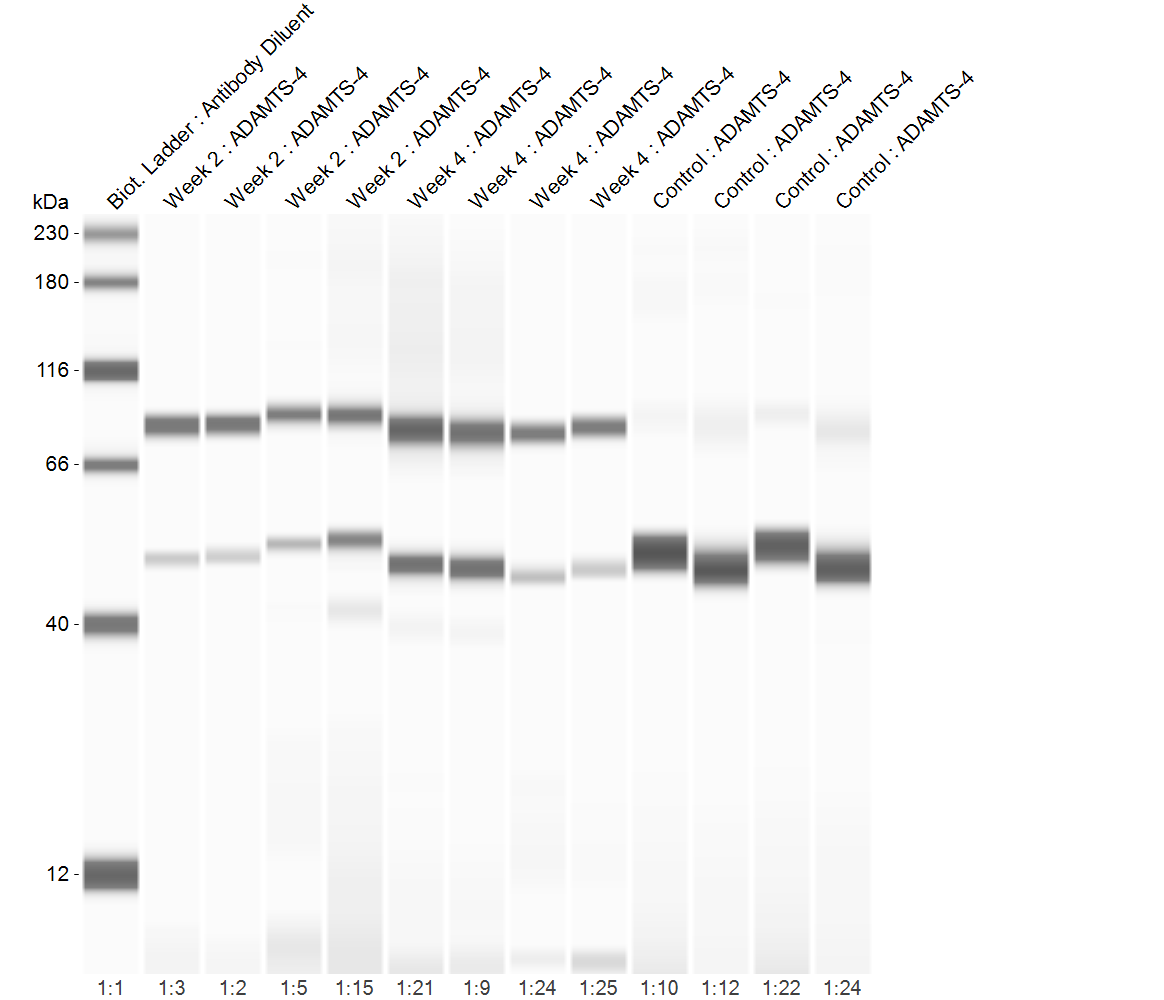
****Whole Blot Images***

Ad Figure 3A. ***Ex vivo* quantification of ADAMTS4, Galectin-3, and α-smooth-muscle-actin expression in aneurysmal and control aortas.**

Whole blot image for *a Disintegrin and Metalloproteinase with Thrombospondin Motifs 4* (ADAMTS4), showing protein expression of one sample per animal with abdominal aortic aneurysm as well as control animals.


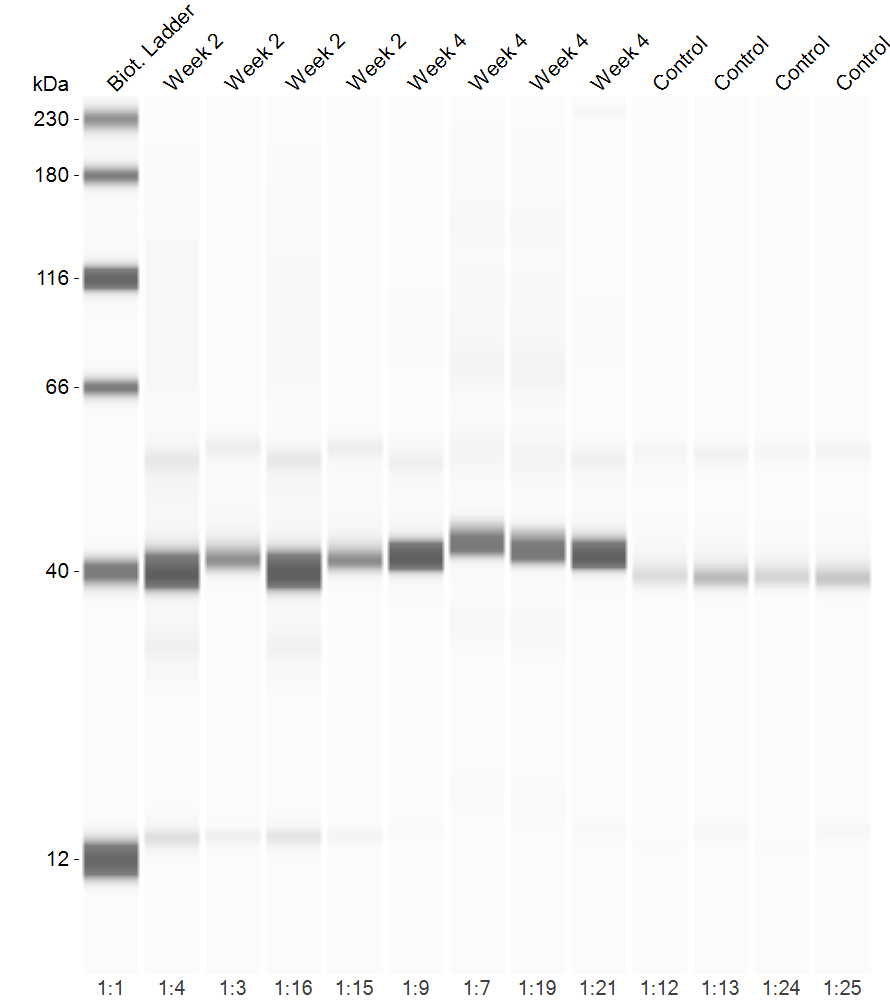
Ad Figure 3B. ***Ex vivo* quantification of ADAMTS4, Galectin-3, and α-smooth-muscle-actin expression in aneurysmal and control aortas.**

Whole blot image for Galectin-3, showing protein expression of one sample per animal with abdominal aortic aneurysm as well as control animals.
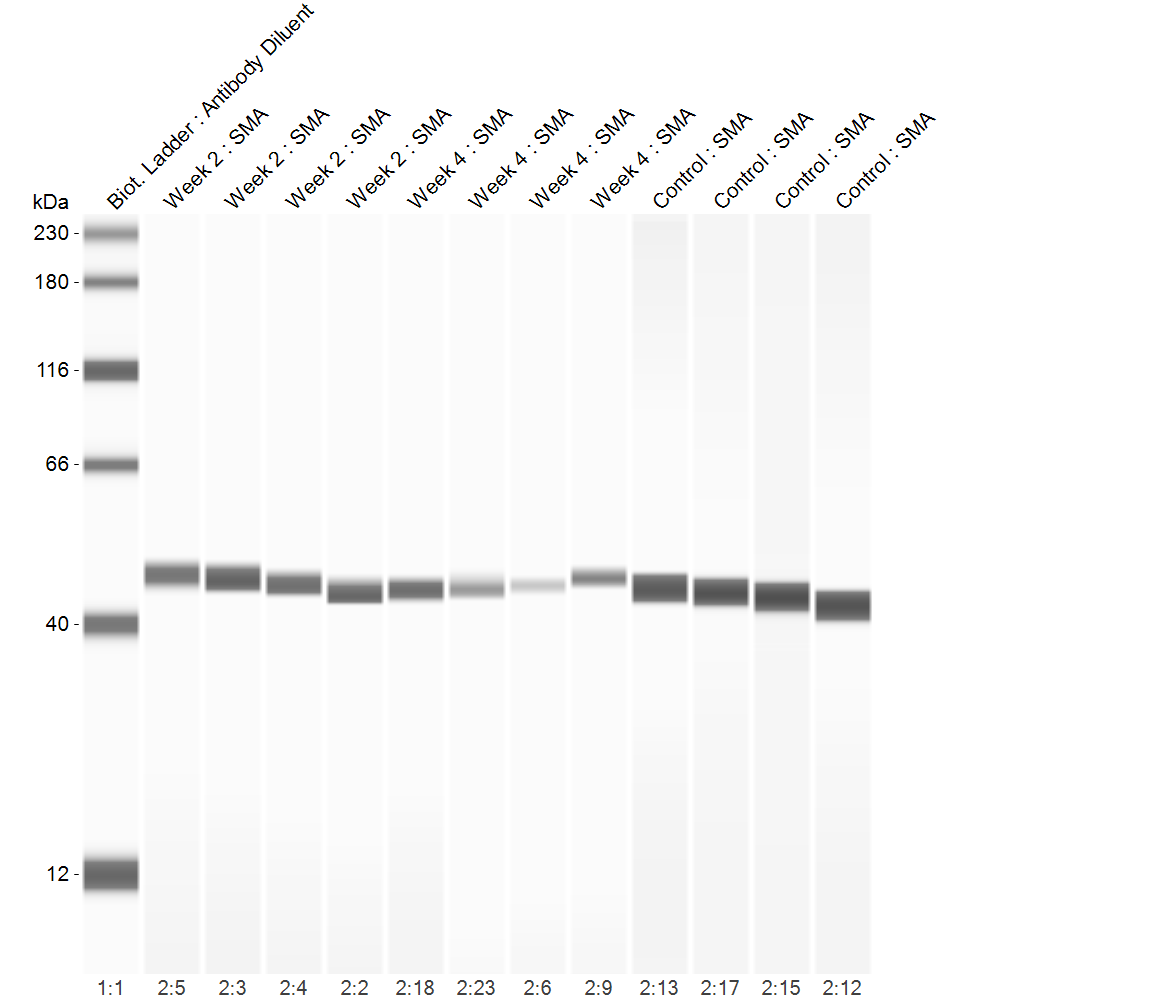
Ad Figure 3C. ***Ex vivo* quantification of ADAMTS4, Galectin-3, and α-smooth-muscle-actin expression in aneurysmal and control aortas.**

Whole blot image for α-Smooth-Muscle-Actin (SMA), showing protein expression of one sample per animal with abdominal aortic aneurysm as well as control animals.

**Supplementary Information**

***Animal Housing Specifications***

Animal experiments

The experiments were carried out by a veterinarian and all possible steps were taken to avoid animal suffering. Experiments were performed according to the guidelines and regulations of the Federation of Laboratory Animal Science Associations (FELASA) and the local Guidelines and Provisions for Implementation of the Animal Welfare Act, State Office of Health and Social Affairs. Pigs were acquired in groups of three between February 2023 and February 2024 from the Bundes Hybrid Zuchtprogramm, Dahlenburg-Ellringen, Germany, and Gerd Heinrichs, Heinsberg, Germany.

Upon arrival at the animal facility, the pigs were weighed and clinically examined. They were housed in a fully tiled pen measuring 12.86 m², which could be divided into two separate pens for training sessions. Straw and sawdust were provided as bedding. The animals were exposed to natural daylight and additional artificial lighting from 6 AM to 6 PM. The ambient temperature was maintained at 20 °C ± 2 °C, with relative humidity at 55 % ± 10 %. Water was available ad libitum through two self-drinkers.

The diet consisted of ‘Pig feed 1 [>35 kg LM] energy-reduced 4 mm’ pellets from Ssniff, Soest, Germany. The animals received 70 g of pellets per kilogram of body weight per day, divided into two meals. Hay was provided as a dietary supplement. During training, food rewards such as banana mash, apples, and canned dog food were used. For enrichment, chains, balls, and rubber toys were permanently available. The enclosure was cleaned daily, with all food leftovers and bedding material replaced.

**Supplementary Material**

***Endovascular Interventional Protocol Medications and Material***

**Atropine (Atropinsulfate 0.5 mg/mL):** #00648037; B. Braun Melsungen AG, Melsungen, Germany.

**Azaperone (Stresnil 40 mg/mL Solution)**: #BE-V000673; Elanco GmbH, Cuxhaven, Germany.

**Ketamine (100 mg/ml)**: #1200; CP‑Pharma HGmbH, Burgdorf, Germany.

**Xylazine (Xylavet® 100 mg/mL)**: #401510.01; CP‑Pharma HGmbH, Burgdorf, Germany.

**Propofol (1% 10 mg/1 ml MCT)**: #16661502; Fresenius Kabi Deutschland GmbH, Bad Homburg v. d. Höhe, Germany.

**Propofol (2% 20 mg/1 ml MCT):** #00194346; Fresenius Kabi Deutschland GmbH, Bad Homburg v. d. Höhe, Germany.

**Fentanyl i.v. Solution (Fentanyl‑Hameln 50 µg/mL):** #06143410; Hameln Pharma GmbH, Hameln, Germany.

**Isoflurane (Isoflurane CP 1 mg/mL):** #1214; CP‑Pharma HGmbH, Burgdorf, Germany.

**Sterofundin® ISO:** #2506375; B. Braun Melsungen AG, Melsungen, Germany.

**Noradrenaline (Noradrenaline Kabi 1 mg/mL):** #7000317.00.00; Fresenius Kabi Deutschland GmbH, Bad Homburg v. d. Höhe, Germany.

**Glucose (Glucose B. Braun Vet Care 5 g/100 mL):** #V7006055.00.0; B. Braun Melsungen AG, Melsungen, Germany.

**Potassium Chloride (Kalium Chlorid 7.45 % MPC Conc. Inj.):** #03140598; B. Braun Melsungen AG, Melsungen, Germany.

**Fentanyl Patches (Fentanyl-1A Pharma 50 µg/h Matrixpfl.**): #00682809; 1A Pharma (Novartis AG), Basel, Switzerland.

**Midazolam (Midazolam-ratiopharm® 15 mg/3 mL Inj.):** #44856.01.00; Merckle GmbH, Blaubeuren, Germany.

**Buprenorphine (TEMGESIC® 0.3 mg/mL Ampules):** #997.00.00; EUMEDICA Pharmaceuticals GmbH, Lörrach, Germany.

**Heparin (Heparin-Natrium-5000-ratiopharm®):** #43001343; Merckle GmbH, Blaubeuren, Germany.

**Tromethamine (THAM‑Köhler 3 mmol/mL):** #4699.99.99; Dr. Franz Köhler Chemie GmbH, Bensheim, Germany.

**Metronidazole (Metronidazol Fresenius 500mg/100ml I.v. Solution):** #05105488; Fresenius Kabi Deutschland GmbH, Bad Homburg v. d. Höhe, Germany.

**Metamizole Ampules (Novaminsulfon-ratiopharm® 500 mg/mL Ampules)**: #08713863; Merckle GmbH, Blaubeuren, Germany.

**Metamizole Powder (METAPYRIN® Oral 100 %):** #11182642; Serumwerk Bernburg AG, Bernburg, Germany.

**Sulbactam/Ampicillin (Unacid® 2000 mg/1000 mg)**: #023383‑67987‑100; Pfizer Pharma GmbH, Berlin, Germany.

**Aqua Ad Injectablia (Aqua Inject. Miniplasco® Connect Ampules):** #3113087; B. Braun Melsungen AG, Melsungen, Germany.

**Tris HCl Buffer (1 M Tris‑HCl UltraPure™, pH 8.0):** #15568025; Invitrogen (Thermo Fisher Scientific Inc.), Waltham (MA), USA.

**Collagenase (CLS-1 Collagenase, Type 1):** #LS004194; Worthington Biomedical Corp., Lakewood (NJ), USA.

**Elastase (10 mg ≥200 U/mg):** #39445‑21‑1; Carl Roth GmbH + Co. KG, Karlsruhe, Germany.

**Calcium Chloride:** #10043‑52‑4; Merck KGaA, Darmstadt, Germany.

**PROGREAT® Microcath.:** #MC‑PE28131ZB; Terumo MedicalCorp., Somerset (NJ), USA.

**Foley-Type Urinary Catheter 12F:** #2310014212; Dahlhausen GmbH, Cologne, Germany.

**Percutaneous Entry Thinwall Needle:** 18G #BSDN‑18‑9.0/ 20G #SDN‑20‑4.0; Cook Group Inc., Bloomington (IN), USA.

**Radifocus™ Vessel Dilator:** 6F RF‑VD60K10M/ 8F RF‑VD80K10M; Terumo Medical Corp., Somerset (NJ), USA.

**Radifocus™ Introducer II Standard Kit A:** 6F #RS‑A60K10AQ/ 8F #RS‑A80K10SQ; Terumo Medical Corp., Somerset (NJ), USA.

**TEMPOTM Pigtail Catheter 4F**: #451‑403 L5; Cordis Corp., Hialeah (FL), USA.

**Atlas™ PTA Balloon Catheter (14 mm × 40 mm, 75 cm, 7F)**: #AT75144; Becton Dickinson GmbH, Heidelberg, Germany.

**Fogarty® Arterial Embolectomy Catheters:** 5.5F #12‑080‑5FP/ 6F #12‑080‑6F; Edwards Lifesciences Corp., Irvine (CA), USA.

**ANGIO‑SEAL® VIP Vascular Closure Device:** 6F #610130/ 8F #610131; Terumo Medical Corp., Somerset (NJ), USA.

***Ultrasound Examinations***

**Ultrasound Gel:** #4251765100672; medimex GmbH, Limburg a. d. Lahn, Germany.

**Ultrasound system:** ArtUs EXT-2H; Telemed Ltd., Vilnius, Lithuania.

**Transducer/probe:** C5-2H60-A5; Telemed Ltd., Vilnius, Lithuania.

**Acquisition software:** Echo Wave II, Version 4.2.0 (64-bit); Telemed Ltd., Vilnius, Lithuania.

**Analysis software:** Horos, Version 4.0.1; The Horos Project, Annapolis, MD, USA.

***ADAMTS4-Specific Gadolinium-Bound Probe***

**Fmoc-protected amino acids (various):** various; Gyros Protein Technologies AB, Tucson (AZ), USA.

**Oxyma Pure:** #ACT-25-OXP / #ACT-100-OXP / #ACT-1K-OXP; Gyros Protein Technologies AB, Tucson (AZ), USA.

**N,N′-Diisopropylcarbodiimide (DIC):** #693-13-0; Carbolution Chemicals GmbH, St. Ingbert, Germany.

**N,N-Dimethylformamide (DMF), for synthesis:** #6251.1; Carl Roth GmbH, Karlsruhe, Germany.

**Ethyl acetate, ROTISOLV® HPLC:** #7336.1 / #7336.2; Carl Roth GmbH, Karlsruhe, Germany.

**Diethyl ether, for synthesis:** #5920.2; Carl Roth GmbH, Karlsruhe, Germany.

**Dimethyl sulfoxide (DMSO), for synthesis:** #7029.1; Carl Roth GmbH, Karlsruhe, Germany.

**Acetic anhydride, ROTIPURAN® ≥99 %:** #CP28.1; Carl Roth GmbH, Karlsruhe, Germany.

**Acetonitrile, ROTISOLV® ≥99.9 % HPLC Gradient Grade:** #8825.2; Carl Roth GmbH, Karlsruhe, Germany.

**Piperidine (Fmoc deprotection grade):** #SOL-010; Iris Biotech GmbH, Marktredwitz, Germany.

**Trifluoroacetic acid (TFA):** #SOL-011; Iris Biotech GmbH, Marktredwitz, Germany.

**Rink Amide AM resin (≈0.64 mmol/g):** #BR-1340; Iris Biotech GmbH, Marktredwitz, Germany.

**DOTA(tBu)₃–OH (DOTA-tris(tBu)ester):** #10-F792189; Fluorochem EU Ltd., Cork, Ireland.

**Gadolinium(III) chloride hydrate (GdCl₃·H₂O):** #AB120502; abcr GmbH, Karlsruhe, Germany.

**PurePep® Sonata+ peptide synthesizer:** Gyros Protein Technologies AB, Tucson (AZ), USA / Uppsala, Sweden.

**PurePep® EasyClean (PEC) Linker RC+ / Kits:** #P0020873 (Starter Kit 8×25 µmol) / #P0020872 (Starter Kit 8×100 µmol); Gyros Protein Technologies AB, Uppsala, Sweden.

**Waters ACQUITY UPLC H-Class system (UPLC-ESI-MS):** Waters Corp., Milford (MA), USA.

**C18 column (ACQUITY UPLC BEH C18, 1.7 µm, 2.1 × 50 mm):** #186002350; Waters Corp., Milford (MA), USA.

**NaCl 0,9 % Miniplasco connect Ampules:** #2350756; B. Braun Melsungen AG, Melsungen, Germany.

**Millex^™^ sterile syringe filter, pore size 0.22 μm, diam. 33 mm, sterile, hydrophilic**: # SLGSR33SS; Merck Millipore, Billerica (MA), USA.

***MR Imaging Procedure and Analysis***

**Propofol (1% 10 mg/1 ml MCT):** #16661502; Fresenius Kabi Deutschland GmbH, Bad Homburg v. d. Höhe, Germany.

**Propofol (2% 20 mg/1 ml MCT):** #00194346; Fresenius Kabi Deutschland GmbH, Bad Homburg v. d. Höhe, Germany.

**Biograph mMR 3 T whole-body MR scanner:** Siemens Healthineers AG, Erlangen, Germany.

**mMR Body Coil:** RF transmit/receive abdominal coil), **#10606492**; Siemens Healthineers AG, Erlangen, Germany.

**Horos software:** Version 4.0.1; The Horos Project, Annapolis, MD, USA.

***Harvest Procedure and Histopathology***

**Tissue‑Tek O.C.T. Compound:** #4583; Sakura Finetek USA, Torrance (CA), USA.

**MorFFFix® Formaldehyde‑Subst.:** #13616; MORPHISTO GmbH, Offenbach, Germany.

**Verhoeff van Gieson Staining Kit:** #12739; MORPHISTO GmbH, Offenbach, Germany.

**Picro‑Sirius Red Staining Kit:** #13425; MORPHISTO GmbH, Offenbach, Germany.

**Von Kossa Staining Kit:** #ab150687; Abcam Ltd., Cambridge (EN), UK.

**Acetone ≥99 %:** #L10407.0F; Thermo Fisher Scientific Inc., Waltham (MA), USA.

**Phosphate Buffered Saline, pH 7.4:** #P4417; Sigma‑Aldrich® (Merck KGaA), Darmstadt, Germany.

**Keyence BZ-X800 microscope:** Keyence Corp., Osaka, Japan.

**ImageJ software:** Version 1.54f; National Institutes of Health, Bethesda, MD, USA.

***Immunofluorescence Staining***

**Antibody Diluent, Background Reducing):** #S3022; Agilent Technologies, Santa Clara (CA), USA.

**DAPI Staining Solution (ROTI® Mount FluorCare DAPI):** #HP19.1; Carl Roth GmbH + Co. KG, Karlsruhe, Germany.

**Goat Anti-Mouse IgG (H+L) Highly Cross-Adsorbed Secondary Antibody, Alexa Fluor™ 647:** #A-21236; Invitrogen, Thermo Fisher Scientific Inc., Waltham (MA), USA.

**Donkey Anti-Rabbit IgG (H+L) Highly Cross-Adsorbed Secondary Antibody, Alexa Fluor™ 647:** #A-31573; Invitrogen, Thermo Fisher Scientific Inc., Waltham (MA), USA.

**Galectin-3/Mac2 Antibody:** #14979-1-AP; Proteintech Group Inc., Rosemont (IL), USA.

**α-SMA Antibody:** #sc-53142; Santa Cruz Biotechnology Inc., Dallas (TX), USA.

**10 % Sodium Dodecyl Sulphate:** #2326.1; Carl Roth GmbH + Co. KG, Karlsruhe, Germany.

**Keyence BZ-X800 microscope:** Keyence Corp., Osaka, Japan.

**Keyence Hybrid Cell Count Software:** Version 1.1.1.8; Keyence Corp., Osaka, Japan.

***Western Blotting***

**1 M Tris‑HCl:** #9090.2; Carl Roth GmbH + Co. KG, Karlsruhe, Germany.

**7 M Urea:** #2317.1; Carl Roth GmbH + Co. KG, Karlsruhe, Germany.

**Glycerol:** #G5516; Sigma‑Aldrich® (Merck KGaA), Darmstadt, Germany.

**Halt™ Protease and Phosphatase Inhibitor Cocktail (100X):** #78440; Thermo Fisher Scientific Inc., Waltham (MA), USA.

**Pierce™ BCA Protein Assay Kit:** #23227; Thermo Fisher Scientific Corp., Waltham (MA), USA.

**12–230 kDa Jess™ Separation Module:** #SM-W001; ProteinSimple™ Biotechne Corp., Minneapolis (MN), USA.

**Jess™ Anti-Rabbit Detection Module:** #DM-001; ProteinSimple™ Biotechne Corp., Minneapolis (MN), USA.

**Jess™ Anti-Mouse Detection Module:** #DM-002; ProteinSimple™ Biotechne Corp., Minneapolis (MN), USA.

**Jess™ RePlex™ Module:** #RP-001; ProteinSimple™ Biotechne Corp., Minneapolis (MN), USA.

**Jess™ Total Protein Detection Module:** #DM-TP01; ProteinSimple™ Biotechne Corp., Minneapolis (MN), USA.

**Compass for Simple Western software:** Version 7.0.0, ProteinSimple™ Biotechne Corp., San Jose, CA, USA.

***Laser Ablation-Inductively Coupled-Mass Spectrometry***

**Laser ablation system:** imageBIO266; Elemental Scientific Lasers, Bozeman, MT, USA.

**Dual concentric injector:** Elemental Scientific Lasers, Bozeman, MT, USA.

**iCAP TQ ICP-MS:** Thermo Fisher Scientific, Bremen, Germany.

**Cryostat (Cryostar NX70):** Thermo Fisher Scientific, Waltham, MA, USA.

**Imajar software:** Version 3.64; Robin Schmid, Münster, Germany.

**Gelatin pure:** #4274.1; Carl Roth GmbH + Co. KG, Karlsruhe, Germany.

**Iron(II) sulfate heptahydrate (99.5-105.0 %, AnalaR NORMAPUR®): #24244.232;** VWR Inc., Darmstadt, Germany.

**Gadolinium(III) chloride (99.9 %, trace metal basis, anhydrous)**: **#383560050;** Alfa Aesar, Thermo Fisher Inc., Karlsruhe, Germany.

***Statistical Analyses***

**IBM SPSS Statistics:** Version 29.0.0; IBM Corp., Armonk, NJ, USA.

**GraphPad Prism:** Version 10.4.1; Dotmatics, Boston, MA, USA.

**BioRender:** 2026; Science Suite Inc., Toronto, ON, Canada.**Supplementary** **References**

1. Ranner-Hafferl MLHH, Mangarova DB, Mein J, Heyl JL, Möckel J, Schnapauff D, Auer TA, Collettini F, Kaufmann JO, Adams LC, Makowski MR, Hamm B, Kader A, Brangsch J. An endovascular porcine model of abdominal aortic aneurysm for interventional radiology research. *Eur Radiol Exp*. 2026;10:6. doi:10.1186/s41747-025-00673-z
2. Zitterbart R, Berger N, Reimann O, Noble GT, Ludtke S, Sarma D, Seitz O. Traceless parallel peptide purification by a first-in-class reductively cleavable linker system featuring a safety-release. *Chem Sci*. 2021;12:2389–2396. doi: 10.1039/d0sc06285e
3. Kaufmann JO, Brangsch J, Kader A, Saatz J, Mangarova DB, Zacharias M, Kempf WE, Schwaar T, Ponader M, Adams LC, et al. ADAMTS4-specific MR probe to assess aortic aneurysms in vivo using synthetic peptide libraries. *Nat Commun*. 2022;13:2867. doi:10.1038/s41467-022-30464-8
4. Kashiwagi M, Enghild JJ, Gendron C, Hughes C, Caterson B, Itoh Y, Nagase H. Altered proteolytic activities of ADAMTS-4 expressed by C-terminal processing. *J Biol Chem*. 2004;279:10109–10119. doi: 10.1074/jbc.M312123200
5. Ren P, Hughes M, Krishnamoorthy S, Zou S, Zhang L, Wu D, Zhang C, Curci JA, Coselli JS, Milewicz DM, et al. Critical Role of ADAMTS-4 in the Development of Sporadic Aortic Aneurysm and Dissection in Mice. *Sci Rep*. 2017;7:12351. doi: 10.1038/s41598-017-12248-z
6. Rodríguez LC, CampaTa AMG, Linares CJ, Ceba MR. Estimation of Performance Characteristics of an Analytical Method Using the Data Set Of The Calibration Experiment. *Analytical Letters*. 1993;26:1243–1258. doi: 10.1080/00032719308019900
7. De Nisco G, Chiastra C, Hartman EMJ, Hoogendoorn A, Daemen J, Calo K, Gallo D, Morbiducci U, Wentzel JJ. Comparison of Swine and Human Computational Hemodynamics Models for the Study of Coronary Atherosclerosis. *Front Bioeng Biotechnol*. 2021;9:731924. doi: 10.3389/fbioe.2021.731924
8. Baxter BT, Matsumura J, Curci JA, McBride R, Larson L, Blackwelder W, Lam D, Wijesinha M, Terrin M, Investigators NTC. Effect of Doxycycline on Aneurysm Growth Among Patients With Small Infrarenal Abdominal Aortic Aneurysms: A Randomized Clinical Trial. JAMA. 2020;323:2029–2038. doi:10.1001/jama.2020.5230
9. Meijer CA, Stijnen T, Wasser MN, Hamming JF, van Bockel JH, Lindeman JH, Pharmaceutical Aneurysm Stabilisation Trial Study G. Doxycycline for stabilization of abdominal aortic aneurysms: a randomized trial. Ann Intern Med. 2013;159:815–823. doi:10.7326/0003-4819-159-12-201312170-00007
10. Spears LD, Razani B, Semenkovich CF. Interleukins and atherosclerosis: a dysfunctional family grows. Cell Metab. 2013;18:614–616. doi:10.1016/j.cmet.2013.10.009
11. Mastoraki ST, Toumpoulis IK, Anagnostopoulos CE, Tiniakos D, Papalois A, Chamogeorgakis TP, Angouras DC, Rokkas CK. Treatment with simvastatin inhibits the formation of abdominal aortic aneurysms in rabbits. Ann Vasc Surg. 2012;26:250–258. doi:10.1016/j.avsg.2011.09.003
12. Lu H, Sun J, Liang W, Chang Z, Rom O, Zhao Y, Zhao G, Xiong W, Wang H, Zhu T, et al. Cyclodextrin Prevents Abdominal Aortic Aneurysm via Activation of Vascular Smooth Muscle Cell Transcription Factor EB. Circulation. 2020;142:483–498. doi:10.1161/CIRCULATIONAHA.119.044803
13. Schack AS, Stubbe J, Steffensen LB, Mahmoud H, Laursen MS, Lindholt JS. Intraluminal infusion of Penta-Galloyl Glucose reduces abdominal aortic aneurysm development in the elastase rat model. PLoS One. 2020;15:e0234409. doi:10.1371/journal.pone.0234409
